# Effect of Probiotics on Glycemic Control: A Systematic Review and Meta-Analysis of Randomized, Controlled Trials

**DOI:** 10.1371/journal.pone.0132121

**Published:** 2015-07-10

**Authors:** Yuting Ruan, Jia Sun, Jie He, Fangyao Chen, Rongping Chen, Hong Chen

**Affiliations:** 1 Department of Endocrinology, Zhujiang Hospital, Southern Medical University, Guangzhou, China; 2 The Second Clinical College of Southern Medical University, Guangzhou, China; 3 Department of Biostatistics, School of Public Health and Tropical Medicine, Southern Medical University, Guangzhou, China; University of Chieti, ITALY

## Abstract

**Background:**

Previous clinical trials indicate that probiotic consumption may improve blood glucose control, however, results from randomized trials on glycemic control have been inconsistent.

**Objective:**

To investigate the effects of probiotics on glycemic control in a systematic review and meta-analysis of randomized controlled trials.

**Data Sources:**

PubMed, Embase, Cochrane Library, and Clinicaltrial.gov through October 2014.

**Data Extraction and Synthesis:**

Two independent reviewers extracted relevant data and assessed study quality and risk of bias. Data were pooled using a random-effects model and expressed as mean differences (MD) with 95% CI. Heterogeneity was assessed (Cochran *Q*-statistic) and quantified (*I* ^2^).

**Results:**

Seventeen randomized controlled trials were included, in which 17 fasting blood glucose (*n* = 1105), 11 fasting plasma insulin (*n* = 788), 8 homeostasis model assessment of insulin resistance (*n* = 635) comparisons were reported. Probiotic consumption, compared with placebo, significantly reduced fasting glucose (MD = -0.31 mmol/L; 95% CI 0.56, 0.06; *p* = 0.02), fasting plasma insulin (MD = -1.29 μU/mL; 95% CI -2.17, -0.41; *p* = 0.004), and HOMA-IR (MD = 0.48; 95% CI -0.83, -0.13; *p* = 0.007).

**Conclusions:**

Probiotic consumption may improve glycemic control modestly. Modification of gut microbiota by probiotic supplementation may be a method for preventing and control hyperglycemia in clinical practice.

## Introduction

Abnormal glucose metabolism is causally related to a greater risk of several chronic disorders, including diabetes, obesity, dyslipidemia, and cardiovascular diseases. Blood glucose can be controlled through diet and lifestyle modification to prevent diabetes or related complications and evidence suggests that dietary constituents and supplements such as omega-3 fatty acids [1], dairy products [2], pistachio [3] and coffee [4] can improve glycemic control or reduce an individual’s risk of diabetes.

Probiotics are defined as live microorganisms with potential health benefits for the host if consumed in adequate amounts [5]. Probiotic benefits have been investigated for improving immune function [6], lowering blood pressure [7], and improving lipids [8]. Data from animal models suggest that probiotics can reduce blood glucose and insulin resistance [9]. Interestingly, research shows that gut microbiota are involved in diabetes and metabolic disorders, revealing that diabetic patients have altered gut microbiota compared to non-diabetic counterparts [10]. Probiotics can be used to alter gut microbiota, and their ability to lower glucose is of interest to researchers [11–13]. However, human clinical trials of probiotics and glucose have yielded mixed results. For instance, some studies indicate that probiotic yogurt ingestion for 6 weeks can significantly improve glucose [12], whereas other studies concluded that this approach had no meaningful effects [14, 15]. Such inconsistent findings complicate approaches to and conclusions about probiotic use. In order to provide better evidence-based guidance on the role of probiotics on glycemic control, a systematic review and meta-analysis of randomized controlled trials (RCTs) was performed to assess the effect of probiotics on the endpoints of fasting glucose, fasting insulin, and homeostasis model assessment of insulin resistance (HOMA-IR).

## Materials and Methods

### 1. Literature search

The online databases PubMed, The Cochrane Library, EMBASE, and Clinicaltrial.gov were searched until October 2014 for relevant studies. The following terms were used to search for relevant publications: ‘probiotic’, ‘lactobacilli’, ‘bifidobacter’, ‘bacillus’, ‘saccharomyces’, ‘enterococcus’, ‘streptococcus’, ‘yogurt’, ‘yoghurt’, ‘sour milk’, ‘fermented milk’, ‘gut microbiota’ in combination with ‘glucose’, ‘blood sugar’, ‘glycemic’, and ‘hyperglycemia’. We supplemented the literature search by scanning reference lists of relevant articles. The methodology of this systematic review was specified in advance and documented in a protocol that was published in a prospective register of systematic reviews, PROSPERO (www.crd.york.ac.uk/PROSPERO; ref CRD42014014498).

### 2. Study selection

Studies were included if they meet the following criteria: (1) human RCTs, (2) included adults ≥ 18 years-of-age with or without hyperglycemia, (3) use of probiotic products as an intervention group, (4) mean fasting blood glucose (+ SD) were reported for the intervention and control groups, (5) subjects had not received intestinal surgery. Studies were excluded if the total number of probiotic bacteria was not reported, if the probiotic contained prebiotics as the intervention product, or if they were not in English.

**Fig 1:**
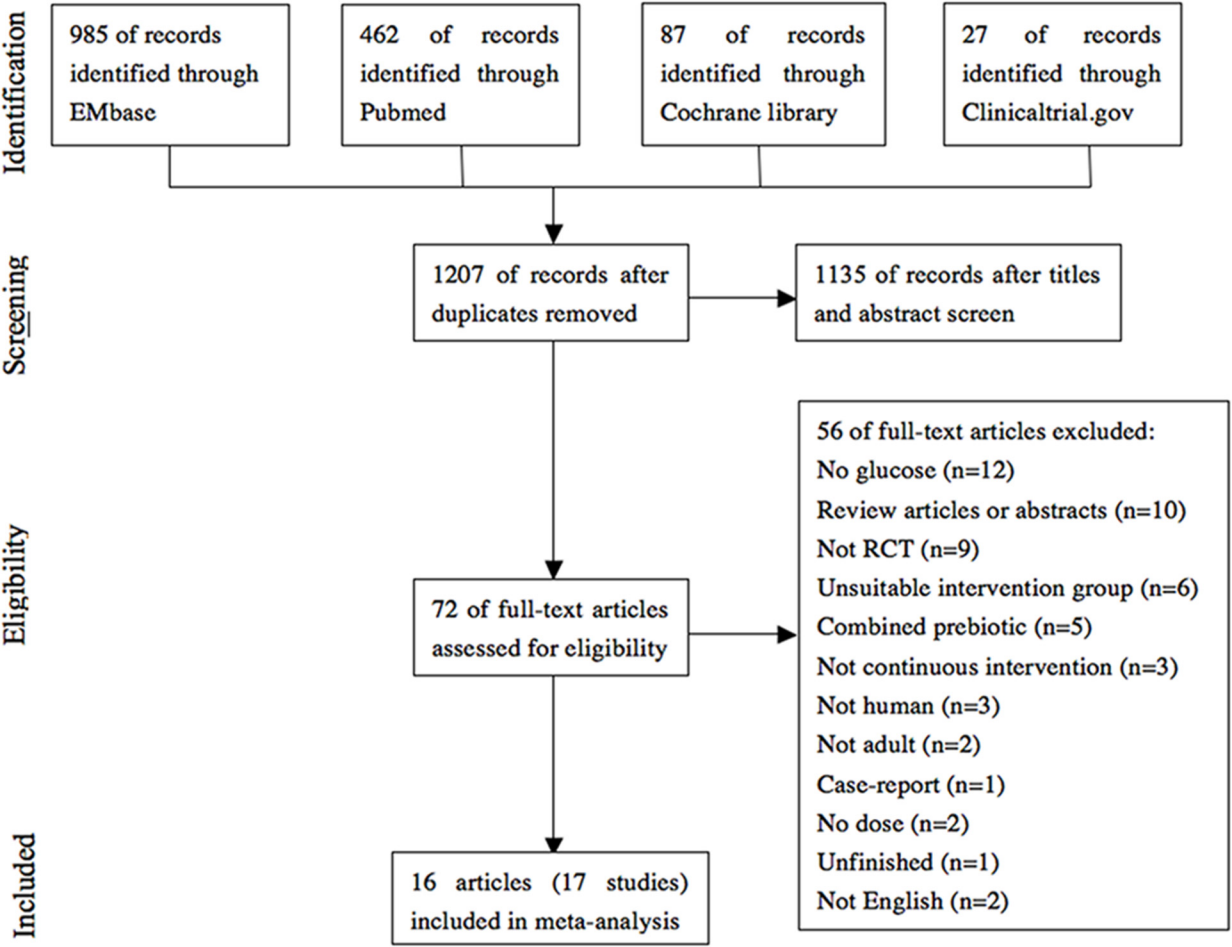
Flowchart for the selection of eligible studies.

YTR and JH conducted an initial screening of studies based on titles and then reviewed abstracts and the full text to assess eligibility criteria independently. Final eligibility was determined through agreement between the 2 reviewers, with any disagreement resolved in consultation with HC. A PRISMA flow chart summarizes these decisions (Fig 1).

### 3. Data extraction

YTR and JH independently extracted these data from eligible publications: probiotics, duration of intervention, sample size, subjects’ characteristics including age, sex, body mass index (BMI), baseline blood glucose, and antidiabetic medication use; probiotics or their fermented dairy products dosage; intervention and treatment results on glucose. We also noted data on baseline and follow-up insulin concentrations and HOMA-IR to measure any correlation between probiotics and glycemic control.

For trials containing multiple intervention arms, a weighted average was applied to combine them in order to create pair-wise comparisons. Ivey and coworkers [14] compared the effect of probiotic in four arms (1 probiotic yoghurt plus probiotic capsules group; 1 probiotic yoghurt plus placebo capsules group; 1 control milk plus probiotic capsules group and 1 control milk plus placebo capsules group). We extracted the data and analyzed subsets separately (probiotic yoghurt plus probiotic capsules group vs. probiotic yoghurt plus placebo capsules group; control milk plus probiotic capsules group vs. control milk plus placebo capsules group). Therefore, 16 articles and 17 RCTs were included in this meta-analysis.

### 4. Data analyses

Statistical analysis was performed according to the *Cochrane Handbook for Statistical Review of Interventions (Version 5*.*0*.*2)*. The difference between the intervention and control arm’s change from baseline value was derived from each trial for endpoints fasting glucose, fasting insulin, and HOMA-IR. Mean differences (MD) of glucose, insulin and HOMA-IR were pooled respectively using a random-effects model due to study heterogeneity. The meta-analysis was performed using RevMan software (Cochrane Review Manager, version 5.2). Statistical tests were two-sided (*p* < 0.05).

Heterogeneity was tested and measured with a *Q*-test and with *I* ^2^ statistics. In general, we regarded heterogeneity as substantial if the *I* ^2^ > 50% or *I* ^2^ > 25% with a low *p* value (< 0.10). We explored sources of heterogeneity by comparing mean differences in each endpoint (fasting glucose, fasting insulin, and HOMA-IR) between subgroups stratified by hyperglycemia, pregnancy, probiotic dose, species, and sources and duration of treatment. To test data robustness, sensitivity analyses were performed in which each individual trial was removed from the meta-analysis and the effect size was recalculated with the remaining trials. Sensitivity analyses also undertook in which small studies (sample size < 20 for each group) were removed from the meta-analysis.

Risk of bias was assessed using the Cochrane Risk of Bias tool (S1 Fig). Potential publication bias was assessed using visual inspection of funnel plots and quantitatively assessed using egger’s tests performed by STATA 10.0 software, where a *p*-value < 0.10 was considered evidence of small study effects.

## Results

### 1. Characteristics of Included Studies

Seventeen clinical trials involving 1,105 participants (551 probiotics, 554 control) were included and these trials were parallel RCTs that were similar with regard to baseline characteristics, indicating successful randomization. Sixteen studies were double-blind design [12, 14–27]; and one was a single-blind design [28]. Seventeen trials reported data for fasting glucose (n = 1105), 11 for fasting insulin (n = 788), and 8 for HOMA-IR (n = 635). In twelve trials, dropout reasons and numbers were noted [12, 14, 16, 18–21, 25–28].

Table 1 displays the characteristics of the included trials. The average baseline fasting blood glucose (FBG) across the studies was 5.89 mmol/L in probiotic group and 5.83 mmol/L in the control group. In five trials [12, 16, 22, 25, 27], patients used antidiabetic medications but they did not change their medications during the study. One of the five studies was used antidiabetic medication (metformin) to treat non-alcoholic steatohepatitis patients, while others were included T2DM patients. The duration of the studies ranged from 3 to 24 weeks and nutrition intake was measured in 7 studies [12, 16, 20–22, 25, 28]; no differences in energy or nutrient intake between intervention and control groups were found. The remainder of the studies only reported that participants were advised to maintain their diet, except for 2 studies in which subjects were instructed to modify dietary intake in both groups [20, 26]. Probiotic species and dose used varied between studies. Eight studies used a single species of probiotics, whereas the others used a combination of equal or more than 2 species. All studies reported good compliance with no side effects from consuming probiotics, except 2 studies that reported subject flatulence, loose stools or constipation [18, 20].

**Table 1:** Characteristics of included studies.

Study	Design, Location	Probiotic Source	Duration (weeks)	Participant, Age (No. of Intervention/No. of Control)	Baseline Characteristics				Probiotic	Dose, CFU	Antidiabetic Medication Use
					*Glucose (mmol/l)*	*BMI (kg/m* ^*2*^ *)*	*Insulin (μU/ml)*	*HOMA-IR*			
Asemi *et al*. (16)	DB,PC,P, Iran	C	8	T2DM, 35–70, (27/27)	7.73	30.89	5.76	2.01	*L*. *acidophilus*, *L*. *rhamnosus*, *L*. *casei*, *L*. *bulgaricus*, *B*. *longum*, *S*. *thermophilus*	3.92×10^10^	YES
Asemi *et al*. (28)	SB,PC,P, Iran	Y	9	Pregnant, 18–30, (37/33)	5.21	ND	7.90	1.82	*S*. *thermophilus*, *L*. *bulgaricus*, *L*. *acidophilus*, *B*. *animalis*	1×10^7^	NO
Bukowska *et al*. (17)	DB,PC,P, Poland	Fermented oatmeal soups	6	HC, men, 40–45, (15/15)	5.94	26.25	ND	ND	*L*. *plantarum*	1×10^10^	NO
Ejtahed *et al*. (12)	DB,PC,P, Iran	Y	6	T2DM, 30–60, (30/30)	7.71	29.05	6.89	ND	*L*. *acidophilus*, *B*. *lactis*	3.98×10^9^	YES
Ivey *et al*. (14)^a^	DB,PC,P, Australia	C, Y	6	OB, 56–77, (40/37)	5.58	30.41	9.79	2.47	*L*. *acidophilus*, *B*. *animalis subsp lactis*	6×10^9^	NO
Ivey *et al*. (14)^b^	DB,PC,P, Australia	C	6	OB, 56–77, (40/39)	5.47	30.80	9.88	2.44	*L*. *acidophilus*, *B*. *animalis subsp lactis*	3×10^9^	NO
Jones *et al*. (18)	DB,PC,P, Canada	C	9	HC, 20–75, (62/62)	5.35	27.30	ND	ND	*L*. *reuteri*	5.8×10^9^	NO
Jung *et al*. (19)	DB,PC,P, Korea	C	6	OB, 19–60, (22/28)	5.75	29.16	10.21	ND	*L*. *Gasseri*	6×10^10^	NO
Laitinen *et al*. (20)	DB,PC,P, Finland	C	20	Pregnant, 25–35, (66/70)	4.53	ND	5.67	1.17	*L*. *rhamnosus*, *B*. *lactis*	1×10^10^	NO
Lindsay *et al*. (21)	DB,PC,P, Ireland	C	4	OB, pregnant, 31–36, (63/75)	4.73	33.55	15.36	3.27	*L*. *salivarius*	1×10^9^	NO
Mohamadshahi al (22)	DB,PC,P, Iran	Y	8	OB, T2DM, 42–59, (20/20)	10.07	28.79	ND	ND	*L*. *Bb12*, *L*. *acidophilus*	1.11×10^9^	YES
Naruszewicz *et al*. (15)	DB,PC,P, Sweden	D	6	Healthy smoker, 35–45, (18/18)	5.89	25.3	9.7	ND	*L*. *plantarum*	2×10^10^	NO
Rajkumar *et al*. (24)	DB,PC,P, India	C	6	OB, 40–60, (15/15)	4.93	28.79	18.15	3.95	*L*. *acidophilus*, *L*. *paracasei*, *L*. *delbrueckii*, *L*. *plantarum*, *B*. *longum*, *B*. *infantis*, *B*. *breve*	1.13×10^11^	NO
Rajkumar *et al*. (23)	DB,PC,P, Japan	C	6	Health, 20–25, (15/15)	4.70	22.53	18.77	3.80	*L*. *salivarius*	4×10^9^	NO
Shavakhi *et al*. (27)	DB,PC,P, Iran	C	24	NASH, 18–75, (31/32)	5.52	28.40	ND	ND	*L*. *acidophilus*,* L*. *rhamnosus L*. *casei*, *L*. *bulgaricus*, *L*. *rhamnosus*, *L*. *bulgaricus*	9.5×10^8^	YES
Shakeri *et al*. (25)	DB,PC,P, Iran	Bread	8	T2DM, 35–70, (26/26)	8.27	30.05	ND	ND	*L*. *sporogenes*	1.30×10^10^	YES
Sharafedtinov *et al*. (26)	DB,PC,P, Estonia	Cheese	3	Met.S, 30–69, (25/11)	7.06	37.27	ND	ND	*L*. *plantarum*	7.5×10^12^	NO

C: capsule; Y: yogurt; D: drink; CFU, colony-forming unit; DB, double blind; HC, hypercholesterolemia; Met.S, metabolic syndrome; NASH, non-alcoholic steatohepatitis; OB, obesity; P, parallel; PC, placebo control; SB, single blind; T2DM, type 2 diabetes mellitus.

### 2. Fasting Blood Glucose

**Fig 2:**
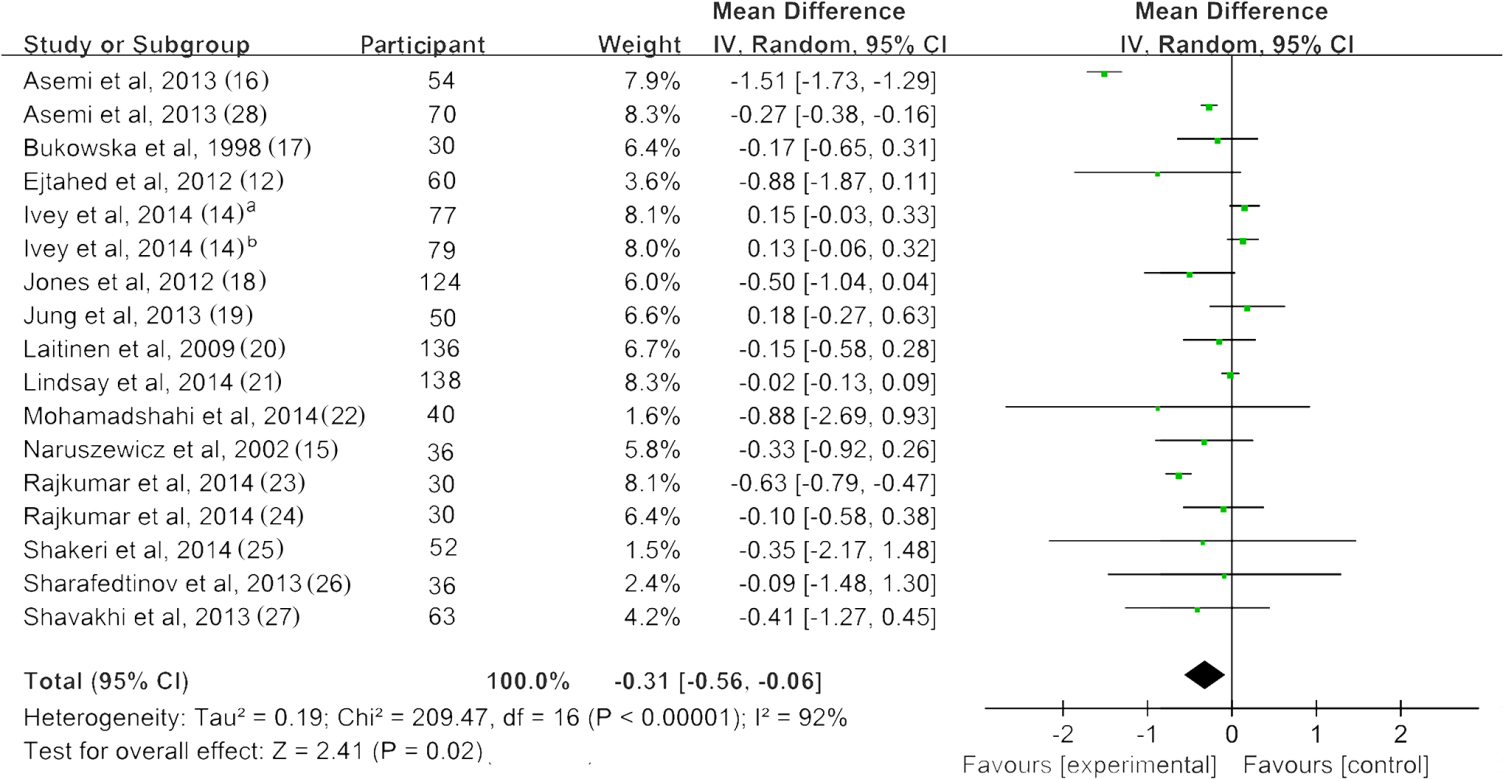
Forest plot of randomized controlled trials comparing the effect of probiotics on fasting blood glucose with placebo/comparator. Weighted mean differences (95% CIs) for fasting blood glucose are shown. Pooled estimates (*diamonds*) calculated by the random effects method. IV, inverse variance.

Fig 2 shows a forest plot of the pooled effect of probiotics on fasting blood glucose. All studies reported changes in fasting blood glucose (FBG). Of the seventeen trials, four studies reported a significant reduction of FBG after probiotic intervention, with mean differences ranging from -0.15 to -1.51 mmol/L [12, 16, 20, 24]. Our meta-analysis of 17 trials indicated a significant reduction of FBG of 0.31 mmol/L (95% CI: 0.56, 0.06; *p* = 0.02) compared with control groups. However, significant evidence of inter-study heterogeneity was observed across studies (*I* ^2^ = 92%, *p* < 0.01).

**Fig 3:**
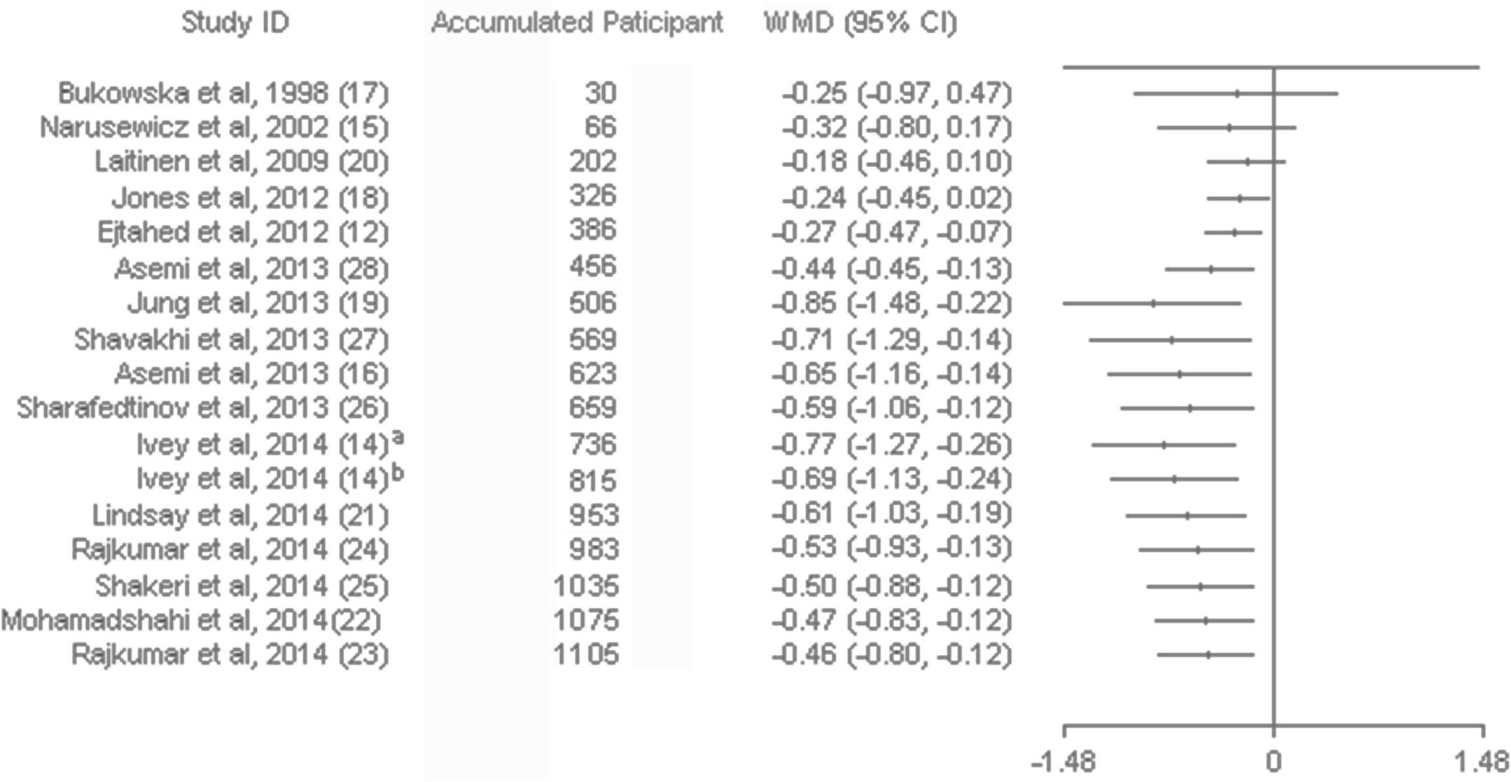
Cumulative Meta-analysis of the probiotics for fasting blood glucose. Error bars indicate the 95% CI of the cumulative meta-analysis estimates as randomized patients accumulate through time. WMD, weight mean difference.

Sensitivity analysis of systematically removing individual trials showed that the removal of four trials with high heterogeneity [14, 16, 24] revealed significance in the overall effect as well (MD = -0.16 mmol/L, *p* < 0.01). Sensitivity analysis also showed that removing studies with small sample sizes (*n* < 20 for each group) [15, 17, 23, 24, 26] did not change the significance of the pooled effect (*p* = 0.04) (S1 Table). Subgroup analysis of studies with hyperglycemic patients revealed a significant reduction of FBG, and these results were not reported in normoglycemic patients. The effect of probiotic on glucose was significant only in the using antidiabetic medications subgroup; however, the non-taking antidiabetic medications subgroup had substantially higher heterogeneity. For trials that included multispecies probiotics revealed significant reduction of glucose while no effect was observed in the single species of probiotic. Trials of > 8 weeks showed a significant effect on glucose reduction. Although significant advantages for intervention ≤ 8 weeks were not observed, there was trend of glucose lowing effect for trials of ≤ 8 weeks (*p* = 0.06). There was no solid evidence for an association between treatment effect of probiotics and the daily dose, source of probiotics or pregnant status (Table 2). A cumulative meta-analysis for FBG indicated that the combined mean difference has tended to be stable since 2013, lingering around a small effect between -0.4 to -0.5. The trend indicated a positive effect of probiotics along time (Fig 3).

**Table 2:** Result of subgroup analysis of included randomized, controlled trials in meta-analysis.

*Groups*	*Fasting blood glucose (mmol/L)*						*Insulin (μU/ml)*					*HOMA-IR*			
	*n*	*WMD (95%CI)*	*P*	*I* ^*2*^ *(%)*	*P*_*heterogeneity*_	*n*	*WMD (95%CI)*	*P*	*I* ^*2*^ *(%)*	*P*_*heterogeneity*_	*n*	*WMD (95%CI)*	*P*	*I* ^*2*^ *(%)*	*P*_*heterogeneity*_
Hyperglycemic															
YES	4	-1.46 (-1.67, -1.25)	<0.01	11	0.34	2	-2.01 (-2.46, -1.56)	<0.01	36	0.21	1	-1.60 (-1.87, -1.33)	<0.01		
NO	13	-0.15 (-0.33, 0.02)	0.09	82	<0.01	9	-1.20 (-2.36, -0.03)	0.05	91	<0.01	7	-0.32 (-0.65, 0.00)	0.05	91	<0.01
Use of antidiabetic medications															
YES	5	-0.98 (-1.58, -0.37)	<0.01	54	0.07	2	-2.01 (-2.46, -1.56)	<0.01	36	0.21	1	-1.60 (-1.87, -1.33)	<0.01		
NO	12	-0.14 (-0.32, 0.04)	0.12	82	<0.01	9	-1.20 (-2.36, -0.03)	0.05	91	<0.01	7	-0.32 (-0.65, 0.00)	0.05	91	<0.01
Pregnant participant															
YES	3	-0.15 (-0.35, 0.05)	0.16	81	<0.01	3	-1.47 (-4.31, 1.38)	0.31	93	<0.01	3	-0.37 (-0.98, 0.24)	0.23	93	<0.01
NO	14	-0.36 (-0.74, 0.01)	0.06	93	<0.01	8	-1.15 (-1.78, -0.52)	<0.01	67	<0.01	5	-0.53 (-1.12, 0.06)	0.08	95	<0.01
Species															
Single species	8	-0.05 (-0.14, 0.05)	0.36	0	0.64	4	-0.83 (-2.94, 1.28)	0.44	66	0.03	2	-0.08 (-0.84, 0.69)	0.85	82	0.02
Multispecies	9	-0.44 (-0.83, -0.05)	0.03	96	<0.01	7	-1.46 (-2.51, -0.41)	<0.01	93	<0.01	6	-0.60 (-0.98, -0.22)	<0.01	94	<0.01
Duration															
> 8 weeks	4	-0.27 (-0.37, -0.17)	<0.01	0	0.78	2	-2.90 (-4.88, -0.93)	<0.01	85	0.01	2	-0.67 (-1.15, -0.20)	<0.01	89	<0.01
≤ 8 weeks	13	-0.32 (-0.67, 0.02)	0.06	94	<0.01	9	-0.92 (-1.61, -0.23)	<0.01	73	<0.01	6	-0.40 (-0.96, 0.17)	0.17	95	<0.01
Daily dose															
≥ 10^11^ CFU	2	-0.62 (-0.78, -0.47)	<0.01	0	0.45	1	-1.17 (-1.52, -0.82)	<0.01			1	-0.77 (-0.94, -0.60)	<0.01		
< 10^11^ CFU	15	-0.28 (-0.56, -0.01)	0.04	92	<0.01	10	-1.28(-2.35, -0.21)	0.02	88	<0.01	7	-0.42 (-0.89, 0.04)	0.07	94	<0.01
Source of probiotic															
Capsule	9	-0.34 (-0.74, 0.06)	0.10	96	<0.01	7	-1.18 (-1.81, -0.54)	<0.01	70	<0.01	6	-0.51 (-0.99, -0.03)	0.04	93	<0.01
Others	8	-0.18 (-0.43, 0.07)	0.16	61	0.01	4	-1.84 (-4.54, 0.86)	0.18	92	<0.01	2	-0.35 (-1.46, 0.77)	0.54	96	<0.01
Total	17	-0.31 (-0.56, -0.05)	0.02	92	<0.01	11	-1.29(-2.17, -0.41)	<0.01	90	<0.01	8	-0.48 (-0.83, -0.13)	<0.01	93	<0.01

Data were meta-analyzed by using a random-effects model or fixed-effects model as appropriate and are presented as WMD. Statistical heterogeneity was assessed by using the chi-square test and quantified by using the I^2^ statistic. WMD: Weight mean difference.

### 3. Fasting Plasma Insulin

**Fig 4:**
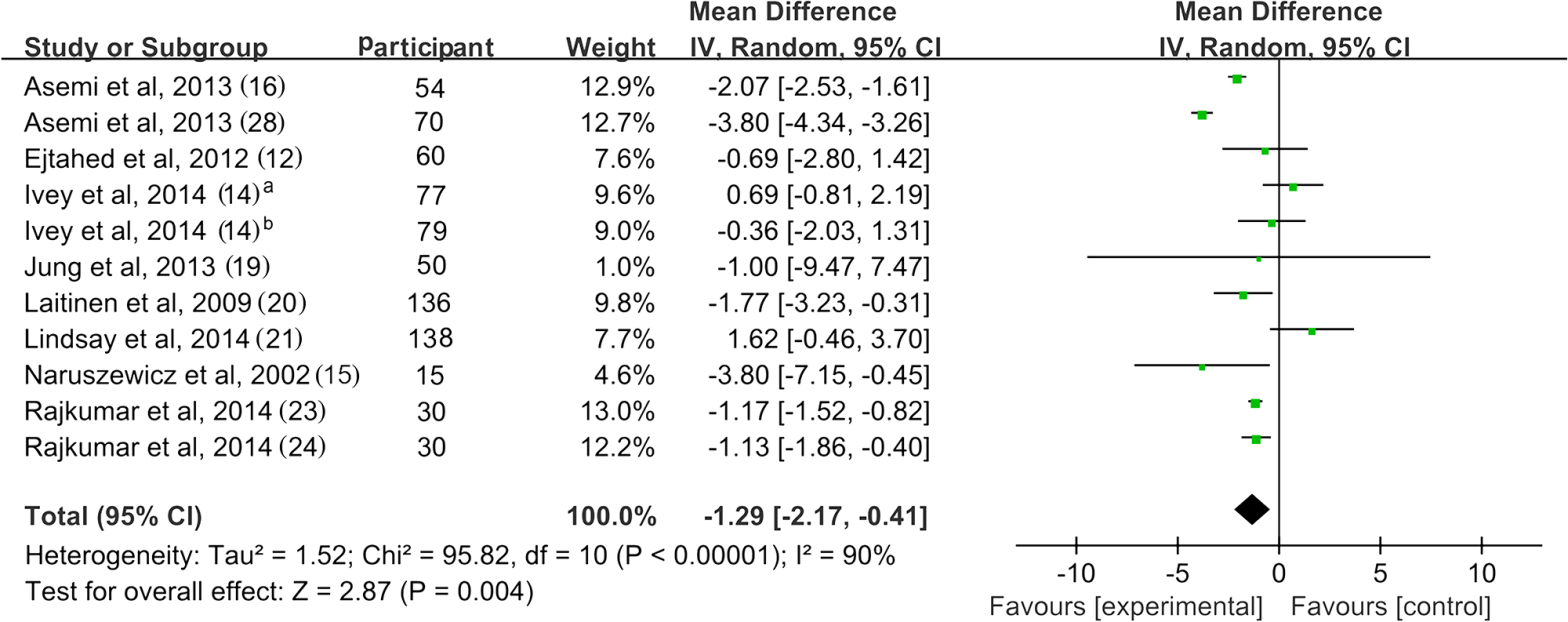
Forest plot of randomized controlled trials comparing the effect of probiotics on fasting plasma insulin with placebo/comparator. Weighted mean differences (95% CIs) for fasting plasma insulin are shown. Pooled estimates (*diamonds*) calculated by the random effects method. IV, inverse variance.

Fig 4 shows a forest plot of the pooled effect of probiotics on fasting plasma insulin. Eleven studies reported the changes in insulin, with 3 studies reporting a significant reduction of insulin after probiotic use [23, 24, 28]. The mean difference ranged from -0.36 to -3.8 μU/mL. The pooled mean difference was -1.29 μU/mL (95% CI -2.17, -0.41; *p* = 0.004) for insulin. Nevertheless, significant inter-study heterogeneity was observed in the overall analysis (*I* ^2^ = 90%, *p* < 0.01).

Sensitivity analysis drew the same conclusion after removing the trials with high heterogeneity [14,16,21,28]. However, removing the trials of small sample size [15,23,24] led to a loss of significance in the overall effect (MD = -1.09 μU/mL; *p* = 0.09) (S1 Table). Subgroup analysis showed that hyperglycemic patients received better effect of insulin reduction than the normoglycemic patients. However, there were only 2 studies included in the hyperglycemic subgroup may result in unreliable conclusions. Subgroup analysis of trials with multispecies probiotics found a significant reduction of insulin. Those trials using a single species of probiotic did not show significant effect compared with control groups. Using capsule as the source of probiotic resulted in significant reduction in insulin; similar results were not found for other sources of probiotics. There was no solid evidence for an association between insulin lowing effect of probiotics and the daily dose or duration due to small number of study and high inter-study heterogeneity (Table 2).

### 4. Homeostasis model assessment of insulin resistance

**Fig 5:**
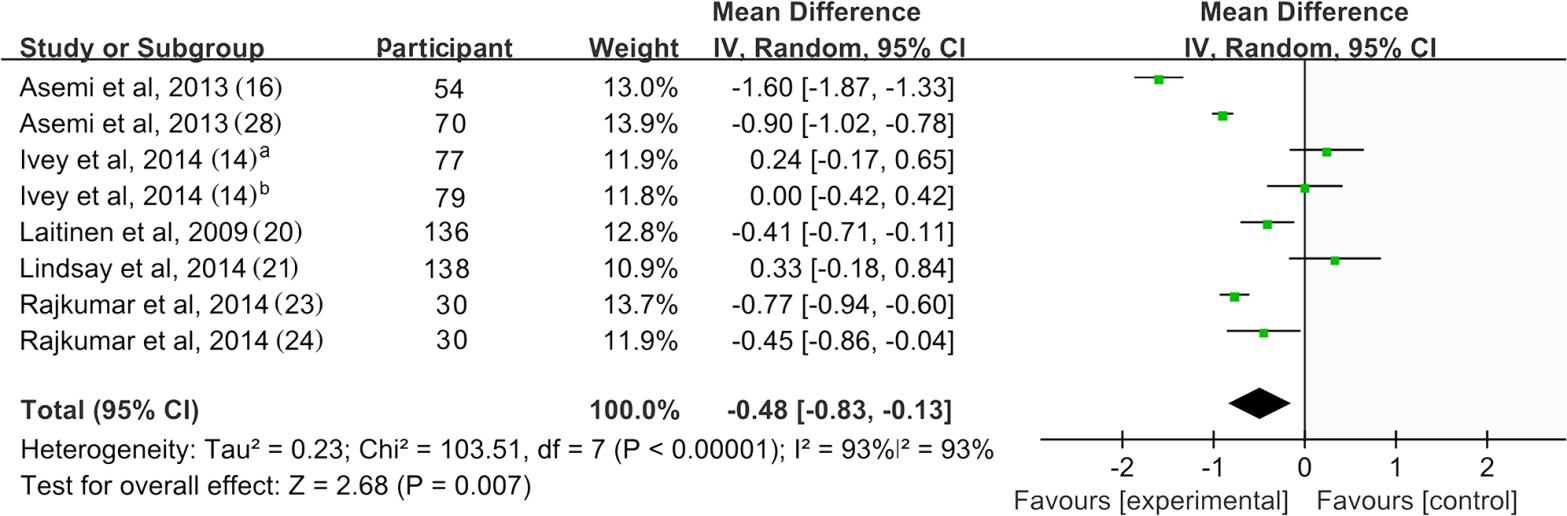
Forest plot of randomized controlled trials comparing the effect of probiotics on HOMA-IR with placebo/comparator. Weighted mean differences (95% CIs) for HOMA-IR are shown. Pooled estimates (*diamonds*) calculated by the random effects method. IV, inverse variance.

Fig 5 shows a forest plot of the pooled effect of probiotics on HOAM-IR. Eight of 17 studies reported changes in HOMA-IR, with 4 studies reporting a significant reduction of HOMA-IR after consuming probiotics[16, 23, 24, 28]. The mean difference ranged from -0.41 to -1.60. The pooled mean difference was -0.48 (95% CI -0.83, -0.13; *p* = 0.007) for HOMA-IR. Significant evidence of inter-study heterogeneity was observed across studies (*I* ^2^ = 93%, *p* < 0.01).

Sensitivity analysis was performed to evaluate the reliability of the pooled mean difference. Results remained consistent after removing the trials with high heterogeneity [14,16,21,23,28]. However, removing the trials of small sample size [23,24] led to a loss of significance in the overall effect (MD = -0.42; *p* = 0.12) (S1 Table). Subgroup analysis of trials with multispecies probiotics found a significant reduction of HOMA-IR. Those trials using a single species of probiotic did not show significant effect compared with control groups. Using capsule as the source of probiotic resulted in significant reduction in HOAM-IR; similar results were not found for other sources of probiotics. There was no solid evidence for an association between HOMA-IR lowing effect of probiotics and the hyperglycemic status, pregnant status, daily dose or duration due to small number of study and high inter-study heterogeneity (Table 2).

### 5. Publication bias

**Fig 6:**
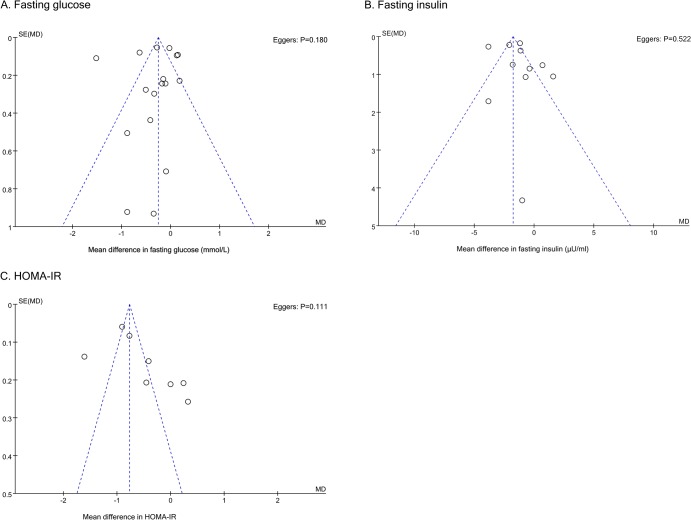
Publication bias funnel plots. Publication bias funnel plots for fasting glucose (A), fasting insulin (B) and HOMA-IR (C). The solid line represents the pooled effect estimate expressed as the weighted mean difference for each analysis. The dashed lines represent pseudo-95% confidence limits. P-values displayed in the top right corner of each funnel plot are derived from quantitative assessment of publication bias by Egger’s test.

Funnel plot and the Egger’s regression were performed to detect potential publication bias. As shown in the Fig 6, funnel plots for fasting glucose and insulin were approximately symmetric while an asymmetry was detected for HOMA-IR. Egger’s regression indicated no significant publication bias for all three indices with p-value equal to 0.180, 0.522 and 0.111.

## Discussion

This is the first study to systematically analyze the effect of probiotics on glycemic control. Overall, probiotics significantly reduced FBG by 0.31 mmol/L, insulin by 1.17 μU/mL and improved HOMA-IR by 0.48, indicating a modest effect of probiotics on glycemic control; however, even small glucose reductions may provide health benefits. Abnormal glucose metabolism carries crucial risks for many metabolic diseases, such as obesity, diabetes, obstructive sleep apnea-hypopnea syndrome (OSAHS), and cardiovascular disease.

The hypothesis that probiotics may be involved in maintenance of healthy gut microbiota and glucose management has received much attention. The ratio of bacteroidetes species in T2DM correlates positively with plasma glucose [29] and alterations in gut microbiota have recently been reported in patients with T2DM, and this may be reversible with probiotic supplement [10]. Dietary supplementation of probiotics for high fructose- and streptozotocin-induced diabetes in rats improved glucose and lipid metabolism, suppressed glucose intolerance and delayed the onset of hyperglycemia, hyperinsulinemia, dyslipidemia, and oxidative stress [30, 31]. Yun’s group [32] found that FBG and 2-hour blood glucose were significantly lower after probiotic ingestion for 3 weeks in db/db mice. In our study, we observed that probiotics had a greater effect on FBG in people with diabetes and there were only trends of glucose-lowing effect in those without diabetes, supporting the notion that probiotics supplementation may generate a greater benefit in individuals with higher FBG levels. We further assessed potential associations of the treatment effect with antidiabetic agents in the subgroup analysis. Four of five trials are included hyperglycemic participants and one included NASH patients. Interestingly, the significant effect on glucose was only observed in those with antidiabetic medications subgroup, which means that it may exist confounding effects on glucose lowing between probiotics and antidiabetic medications. Another explanation may relate to a well understanding phenomenon that the higher baseline glucose levels, the greater reduction with anti-hyperglycemic agents.

There was evidence of substantial inter-study heterogeneity in the overall effect for FBG, insulin and HOMA-IR. Subgroup analysis indicated that diabetes status partially explained the heterogeneity in the overall analysis. Moreover, not all studies reported beneficial effects of probiotics, and thus caution is needed regarding the species, sources and dose to be used, which may have important ramifications on the effects observed and help to explain the heterogeneity across the studies. Subgroup analysis of studies using multispecies of probiotic indicated a more pronounced reduction in FBG, fasting plasma insulin and HOMA-IR; however, it seems to be no effect of probiotics on these endpoints for those trials using a single species of probiotic. The findings of present meta-analysis are in line with the previous studies, both suggesting a combination of probiotic species are more effective than single species products [33]. Although these findings may provide important information for future interventions using probiotics, caution is required because high heterogeneity was observed in the multispecies subgroup. Unfortunately, the lack of trials on species and strains of probiotics made it not practical to analyze the effect of different probiotic species on glycemic control. Secondary, administration of probiotic sources varied among trials with most trials using encapsulated probiotic supplements. Subgroup analysis of studies using the probiotic capsule didn’t reveal significant reductions in FBG compared with other sources. However, an inadequate number of studies that used other sources of probiotics (yogurt, rose-hip drinks, probiotic cheese, etc.) limit these conclusions for the best source of probiotics. In addition, there seems to be no trend between the daily dose of probiotics consumed and change in FBG or insulin or HOMA-IR. However, finding from the subgroup analysis indicate that the reduction in FBG may be greater when the daily dose of probiotics consumption ≥ 10^11^ CFU. This finding may be because of the bias of the low number of trials in the high daily dose subgroup.

Another important observation we made was that the improvements in FBG and HOMA-IR were restricted to trials > 8 weeks, while better insulin reduction was obtained in trials > 8 weeks compared to trials ≤ 8 weeks. However, further studies with longer treatment durations are required to confirm this result because the group with duration > 8 weeks was small with only 2 to 4 studies. Thus, there was no solid evidence for an association between glycemic control of probiotics and the treatment durations. Finally, pregnant women are susceptible to increased insulin resistance and glucose, so a subgroup analysis was conducted to pregnant women. In human clinical trials, supplementation of probiotics combined with dietary counseling has been shown to positively affect glucose metabolism in normoglycemic pregnant women [20]. However, the subgroup analysis of probiotics on FBG, fasting insulin and HOMA-IR was not significant among pregnant women, which might be explained by the inter-individual differences of pregnancy. Furthermore, probiotic strain differences, dose, and treatment duration across different studies might explain differences in outcomes.

How probiotics lower glucose is unclear. They may be related to decreased oxidative stress [12], which is shown to be present in hyperglycemia [34]. Specific strains of lactic acid bacteria have antioxidant properties [35, 36]. For example, Yadav and colleagues [30] reported that probiotic dahi, a fermented milk containing *Lactobacillus acidophilus* and *L*. *casei* delayed the progression of glucose intolerance, hyperglycemia, hyperinsulinemia via decreased oxidative stress in animal models. Also, low-grade chronic inflammation is observed in diabetic and obese individuals and the immune system is crucial for regulation of glucose metabolism. Thus, probiotics may modulate immune responses and systemic low-grade inflammation, in particular by reducing cytokines [37] and suppressing the NF-κB pathway, which mediates immune system microbial activation via toll-like receptors [38]. Laitinen’s group [20] observed pronounced effects of probiotics on reduced glucose and attributed this to immunoregulatory properties. Five of the included studies suggest that the consumption of probiotics decreased inflammatory markers, including hsCRP, IL-6, and TNF-α [15, 16, 22–24]. Also, other studies indicate that systemic inflammation was reduced and intestinal endotoxin (a potential inflammatory stimulant) was decreased with probiotics, lowering insulin resistance and hyperglycemic incidences [39]. Probiotics may attenuate circulating endotoxin, subsequently affecting glucose metabolism [40, 41]

Our work has several limitations. First, we could not obtain data from unpublished literature or non-English published material, which may lead to potential publication bias. However, Egger’s regression showed no significant publication bias, indicating that the unpublished evidence didn’t affect the results of the meta-analysis. Second, some studies had fewer than 20 participants for each experimental group. Forest plots show possible bias, favoring small trials with extreme effects. However, these trials had small weights in our meta-analysis and excluding them only slightly modified probiotic-induced effects on glucose. Third, there was evidence of substantial inter-study heterogeneity in this meta-analysis, which was not explained by most of the priori subgroup analyses. In addition, majority of subgroup analyses were unpowered and it was not possible to assess the effect of other factors that may influence glycemic control due to small number of study.

Thus, our meta-analysis revealed a moderate beneficial effect of probiotics on glycemic control along with lower insulin and HOMA-IR, data that are consistent with a recent meta-analysis suggesting that yogurt intake was associated with an 18% lower risk of T2DM [42]. Modification of gut microbiota by probiotic supplementation may be a method for preventing and control hyperglycemia in clinical practice.

## Supporting Information

S1 Fig Risk of Bias Table of Included Studies. (PDF)

S1 File PRISMA Checklist. (DOCX)

S2 File PROSPERO International Prospective Register of Systematic Reviews. (PDF)

S3 File Revised Manuscript with Track Changes. (DOCX)

S4 File Certification of English Editing. (PDF)

S5 File Reference List of Excluded Articles after Full Text Assessment. (DOCX)

S1 Table Sensitivity Analysis of Included Studies. (DOCX)
