# Supplementary material for: Effect of Probiotics on Glycemic Control: A Systematic Review and Meta-Analysis of Randomized, Controlled Trials
Source: PLoS One. 2015 Jul 10;10(7):e0132121. doi: 10.1371/journal.pone.0132121 (PMC4498615; doi:10.1371/journal.pone.0132121)
Supplement: S1 Fig — (PDF) [file pone.0132121.s001.pdf]

	Random sequence generation (selection bias)	Allocation concealment (selection bias)	Blinding of participants and personnel (performance bias)	Blinding of outcome assessment (detection bias)	Incomplete outcome data (attrition bias)	Selective reporting (reporting bias)	Other bias
Asemi, 2013	+	?	+	+	+	+	+
Asemi, 2013	+	−	−	+	+	+	−
Bukowska, 1998	+	?	?	?	+	+	−
Ejtahed, 2012	+	+	+	+	+	+	−
Ivey, 2014, A	+	+	+	+	+	+	+
Ivey, 2014, B	+	+	+	+	+	+	+
Jones, 2012	+	+	+	+	+	+	+
Jung, 2013	+	?	?	+	?	+	?
Laitinen, 2009	+	+	+	+	+	+	+
Lindsay, 2014	+	+	+	+	+	+	−
Mohamadshahi, 2014	+	?	+	+	+	+	−
Naruszewicz, 2002	+	?	?	+	+	+	+
Rajkumar, 2014	+	+	+	+	+	+	+
Rajkumar, 2014	+	?	+	+	+	+	−
Shakeri, 2014	+	?	+	+	+	+	+
Sharafedtinov, 2013	+	+	+	+	?	+	−
Shavakhi, 2013	+	?	+	+	+	+	+
