# Supplementary material for: Effect of Probiotics on Glycemic Control: A Systematic Review and Meta-Analysis of Randomized, Controlled Trials
Source: PLoS One. 2015 Jul 10;10(7):e0132121. doi: 10.1371/journal.pone.0132121 (PMC4498615; doi:10.1371/journal.pone.0132121)
Supplement: S1 File — (DOCX) [file pone.0132121.s002.docx]

| **Section/topic** | **#** | **Checklist item** | **Reported on page #** |
| --- | --- | --- | --- |
| **TITLE** | | |  |
| Title | 1 | Effect of probiotics on glycaemic control : a systematic review and meta-analysis of randomized, controlled trials | 1 |
| **ABSTRACT** | | |  |
| Structured summary | 2 | ***Aims/hypotheses*** Previous clinical trails have yielded mixed results on the effects of probiotic on blood glucose control. The aim of this systematic review was to address the effects of probiotics on glycaemic control using a meta-analysis of randomized, controlled trails.  ***Methods*** PubMed, Embase, Cochrane Library and Clinicaltrial.gov were searched until October 2014 to identify eligible articles. Meta-analysis using a random-effects model was chosen to analyze the impact of combined trails.  ***Results*** Meta-analysis of seventeen randomized controlled trails involving 1,105 participants showed that probiotic consumption, compared with placebo, produced a significant reduction of 0.31 mmol/l (95% CI 0.56, 0.06; p=0.02) in fasting blood glucose. The pooled mean difference in insulin was -1.17μU/ml (95% CI -2.17, -0.41; *p*=0.004) and mean difference in homeostasis model assessment-insulin resistant (HOMA-IR) was 0.48 (95% CI -0.83, -0.13; *p*=0.007). Subgroup analysis suggested a more significant improvement in glucose in hyperglycemic participants than in normoglycaemic ones. A greater reduction was found with multispecies as compared with single species of probiotic. Duration of intervention ≤8 weeks did not result in a significant reduction in glucose. Furthermore, subgroup analysis of trails with daily dose of probiotics <10^11^ colony-forming units did not present a significant effect on glycaemic control.  ***Conclusions/interpretation*** The present meta-analysis indicates that probiotic consumption may improve glycaemic control by a modest degree, with potentially greater effect when multiple species of probiotic are consumed, the duration is more than 8 weeks, or daily dose is ≥10^11^ colony-forming units.  **Systematic review registration number**: CRD42014014498 | 2-3 |
| **INTRODUCTION** | | |  |
| Rationale | 3 | Abnormal glucose metabolism is causally related to greater risk of several chronic disorders, including diabetes, obesity, dyslipidemia and cardiovascular diseases. Blood glucose can be controlled through diet and lifestyle modification to prevent diabetes or related complications. Probiotics are defined as live microorganisms that may have health benefits for the host if consumed in adequate amounts. The health benefits of probiotic have attracted increasing attention in terms of improving immune system function, blood pressure lowing and improving the lipid profile. Animal models suggest probiotic can reduce blood glucose level and insulin resistance. Novel research shows that the gut microbiota is involved in diabetes and metabolic disorders, revealing that diabetes patients have alterations in the composition of the gut microbiota compared to non-diabetes counterparts. Probiotic is one way of altering the gut microbiota and its glucose-lowing effect has raised much interest in recent years. | 3 |
| Objectives | 4 | The aim of the present meta-analysis is to systematically examine the effect of probiotic on glycaemic control in participants with or without hyperglycemia by conducting a meta-analysis of randomized controlled trials (RCTs). | 4 |
| **METHODS** | | |  |
| Protocol and registration | 5 | The methodology of this systematic review is registered at the International Prospective Register for Systematic Review with the registration number CRD42014014498. The study protocol can be found in the website <http://www.crd.york.ac.uk/PROSPERO> or the supplementary materials | 5 |
| Eligibility criteria | 6 | Studies were included if they met the following inclusion criteria:(1) were human randomized, controlled trials; (2) included adults ≥18 years of age with or without hyperglycemia;(3) used probiotic products as the intervention group; (4) the mean fasting blood glucose, along with standard deviation, were reported for the intervention and control groups; (5) subjects had not undergone intestinal surgery; (6) studies with high methodology equality. Studies were excluded if: (1) the total number of bacteria in the probiotic product used was not reported; (2) the probiotic product contains prebiotics as the intervention product; (3) were not in English. | 5-6 |
| Information sources | 7 | PubMed, The Cochrane Library, EMBASE, Clinicaltrial.gov were searched until October 2014 for relevant studies. We supplemented the literature search by scanning the reference lists of relevant articles. | 5 |
| Search | 8 | The following terms were used to search for relevant publication: ‘probiotic’, ‘lactobacill’, ‘bifidobacter’, ‘bacillus’, ‘saccharomyces’, ‘enterococcus’, ‘streptococcus’, ‘yogurt’, ‘yoghurt’, ‘sour milk’, ‘fermented milk’, ‘gut microbiota’ in combination with ‘glucose’, ‘blood sugar’, ‘glycaemic’, ‘hyperglycemia’. | 5 |
| Study selection | 9 | Two researchers conducted an initial screening of studies based on the titles and then involved a review of abstracts and an examination of the full text in terms of the eligibility criteria. The final eligibility of the articles was determined through agreement between the 2 reviewers, with any disagreement resolved in consultation with a third reviewer. | 6 |
| Data collection process | 10 | Information was carefully extracted from all included publications independently by two of the authors according to the inclusion criteria listed above. Disagreement was resolved by consensus. If these two authors could not reach a consensus, another author was consulted. | 6 |
| Data items | 11 | Two authors independently extracted the data from the publications. The following information will be abstracted from eligible articles: probiotics, duration of intervention, sample size, subjects’ characteristics including age, sex, body mass index (BMI), baseline blood glucose and antidiabetic medication use will also recorded; probiotics or their fermented dairy products dosage; intervention and treatment results on the levels of blood glucose. We also sought data on baseline and follow-up insulin concentrations and HOMA-IR to detect the potential correlation between probiotics and glycaemic control. | 6-7 |
| Risk of bias in individual studies | 12 | The quality of included studies was evaluated independently by two authors according to the Risk of Bias Table of in the *Cochrane Handbook for Systematic Reviews of Interventions.* The risk of bias summary figure was in the supplementary materials. | 6 and S1 Fig. |
| Summary measures | 13 | The principal summary measures are weight mean difference (WMD) of glucose changes between the intervention groups and control groups. | 7 |
| Synthesis of results | 14 | The overall pooled analysis was performed by random-effects model, because an existence of heterogeneity was observed. The heterogeneity was tested and measured with Q-test and I^2^ statistics. In general, we regarded heterogeneity as substantial if the I^2^ was greater than 50% or the I^2^ was greater than 25% with a low *P* value (less than 0.10). | 7-8 |

Page 1 of 2

| **Section/topic** | **#** | **Checklist item** | **Reported on page #** |
| --- | --- | --- | --- |
| Risk of bias across studies | 15 | Potential publication bias was assessed using funnel plots. In case of publication bias, we performed a sensitivity analysis in which smaller studies that reported more extreme effect sizes were excluded. | 8 |
| Additional analyses | 16 | Subgroup analyses were performed by comparing the mean difference in glucose between subgroups stratified by hyperglycemia status, pregnant status, probiotic dose, species, sources and duration of treatment. To test the robustness of the results, we performed sensitivity analyses in which small studies (n<20 for each sample size) are excluded, reanalyzed the data using a fix-effects model after excluding the studies where there may be recognized as high heterogeneity and limited to double-blinding trail. | 7-8 |
| **RESULTS** | | |  |
| Study selection | 17 | A total of sixteen studies included in the meta-analysis. One study reported the results in two subsets. Therefore, seventeen clinical trails involving 1,105 participants (551 probiotics, 554 control) were included in the present analysis. A summary of the review is presented in the PRISMA flow chart. | 8, S1 File. PRISMA Checklist. |
| Study characteristics | 18 | Table 1 shows the studies included in the meta-analysis and their main characteristics. | 9, 11, 12 |
| Risk of bias within studies | 19 | The risk of bias summary figure was in the supplementary materials. The risk of bias results showed that the methodological quality was generally good. | S1 Fig. |
| Results of individual studies | 20 | The main results of individual studies were shown in Figure 2 and 3, respectively. | 13，14 |
| Synthesis of results | 21 | The meta-analysis of 17 trails showed a significant reduction of FBG of 0.31 mmol/l (95% CI 0.56, 0.06; p=0.02) compared with control groups. A high level of heterogeneity was observed across the studies (*I^2^*=92%, p<0.01). The pooled mean difference was -1.29μU/ml (95% CI -2.17, -0.41; p=0.004) for insulin The mean difference ranged from -0.41 to -1.60. The pooled mean difference was -0.48 (95% CI -0.83, -0.13; p=0.007) for HOMA-IR. | 13 |
| Risk of bias across studies | 22 | The funnel plots of studies showed slight asymmetry, which can be interpreted as publication bias. The risk of bias summary figure was in the supplementary materials. | 9 and S2 Fig. |
| Additional analysis | 23 | The result of subgroup analyses and sensitivity analyses was showed in Table 2 and 3, respectively. | 15-17 |
| **DISCUSSION** | | |  |
| Summary of evidence | 24 | Overall, the results showed that consuming probiotics could significantly reduce FBG by 0.31 mmol/l and insulin by 1.17μU/ml and improved HOMA-IR by 0.48, indicating a modest effect of probiotics on glycemic control; however, even a small reduction of glucose might have important public health benefit. Abnormal glucose metabolism is increasing common and carries significant risks of many metabolic diseases, such as obesity, diabetes, OSAHS, and cardiovascular disease. | 17 |
| Limitations | 25 | Discuss limitations at study and outcome level (e.g., risk of bias), and at review-level (e.g., incomplete retrieval of identified research, reporting bias). | 22,23 |
| Conclusions | 26 | In conclusion, the results of this study showed that the consumption of probiotics might improve fasting blood glucose as well as reduce insulin and HOMA-IR. Modification of gut microbiota by probiotic supplementation may be seen as an alternative way to prevent and control hyperglycemia in clinical practice. More randomized, controlled trails with larger sample groups, longer durations are recommended to provide a more definite answer of the effect of different probiotic with different species and doses on glycaemia control. | 23 |
| **FUNDING** | | |  |
| Funding | 27 | None |  |

*From:*  Moher D, Liberati A, Tetzlaff J, Altman DG, The PRISMA Group (2009). Preferred Reporting Items for Systematic Reviews and Meta-Analyses: The PRISMA Statement. PLoS Med 6(6): e1000097. doi:10.1371/journal.pmed1000097

For more information, visit: **www.prisma-statement.org**.

Page 2 of 2
