# Supplementary material for: Effect of Probiotics on Glycemic Control: A Systematic Review and Meta-Analysis of Randomized, Controlled Trials
Source: PLoS One. 2015 Jul 10;10(7):e0132121. doi: 10.1371/journal.pone.0132121 (PMC4498615; doi:10.1371/journal.pone.0132121)
Supplement: S2 File — (PDF) [file pone.0132121.s003.pdf]

PROSPERO International prospective register of systematic reviews

Review title and timescale

1 Review title

Give the working title of the review. This must be in English. Ideally it should state succinctly the interventions or exposures being reviewed and the associated health or social problem being addressed in the review.

Effect of probiotics on glycaemic control: a systematic review and meta-analysis of randomized, controlled trials

2 Original language title

For reviews in languages other than English, this field should be used to enter the title in the language of the review. This will be displayed together with the English language title.

3 Anticipated or actual start date

Give the date when the systematic review commenced, or is expected to commence.

16/07/2014

4 Anticipated completion date

Give the date by which the review is expected to be completed.

30/11/2014

5 Stage of review at time of this submission

Indicate the stage of progress of the review by ticking the relevant boxes. Reviews that have progressed beyond the point of completing data extraction at the time of initial registration are not eligible for inclusion in PROSPERO. This field should be updated when any amendments are made to a published record.

The review has not yet started ×

Review stage	Started	Completed
Preliminary searches	Yes	Yes
Piloting of the study selection process	Yes	Yes
Formal screening of search results against eligibility criteria	Yes	Yes
Data extraction	Yes	Yes
Risk of bias (quality) assessment	Yes	Yes
Data analysis	Yes	Yes

Provide any other relevant information about the stage of the review here.

Review team details

6 Named contact

The named contact acts as the guarantor for the accuracy of the information presented in the register record.

Dr Ruan

7 Named contact email

Enter the electronic mail address of the named contact.

dr_tracy@126.com

8 Named contact address

Enter the full postal address for the named contact.

No.253 industrial avenue, Haizhu District, Guangzhou City, Guangdong Province, China

9 Named contact phone number

Enter the telephone number for the named contact, including international dialing code.

+86-020-62782330

10 Organisational affiliation of the review

Full title of the organisational affiliations for this review, and website address if available. This field may be completed as 'None' if the review is not affiliated to any organisation.

Department of Endocrinology, Zhujiang Hospital, Southern Medical University, Guangzhou, China

Website address:
http://www.zjyy.com.cn/

11 Review team members and their organisational affiliations

Give the title, first name and last name of all members of the team working directly on the review. Give the organisational affiliations of each member of the review team.

Title	First name	Last name	Affiliation
Dr	yuting	Ruan	Department of Endocrinology, Zhujiang Hospital, Southern Medical University, China
Dr	JIA	Sun	Department of Endocrinology, Zhujiang Hospital, Southern Medical University, China
Professor	Hong	Chen	Department of Endocrinology, Zhujiang Hospital, Southern Medical University, China
Dr	Rongping	Chen	Department of Endocrinology, Zhujiang Hospital, Southern Medical University, China
Mr	Jie	He	The Second Clinical College of Southern Medical University, China
Mr	Fangyao	Chen	Department of Biostatistics,School of Public Health and Tropical Medicine, Southern Medical University, Guangzhou, China

12 Funding sources/sponsors

Give details of the individuals, organizations, groups or other legal entities who take responsibility for initiating, managing, sponsoring and/or financing the review. Any unique identification numbers assigned to the review by the individuals or bodies listed should be included.

No external Funding

13 Conflicts of interest

List any conditions that could lead to actual or perceived undue influence on judgements concerning the main topic investigated in the review.

Are there any actual or potential conflicts of interest?

None known

14 Collaborators

Give the name, affiliation and role of any individuals or organisations who are working on the review but who are not listed as review team members.

Title	First name	Last name	Organisation details

Review methods

15 Review question(s)

State the question(s) to be addressed / review objectives. Please complete a separate box for each question.

To systematically review whether the probiotic supplemented can improve biomarkers of glycaemic control.

16 Searches

Give details of the sources to be searched, and any restrictions (e.g. language or publication period). The full search strategy is not required, but may be supplied as a link or attachment.

We will search PubMed, The Cochrane Library, EMBASE, Clinicaltrial.gov databases until October 2014 for relevant studies. To increase the yield of relevant studies, we will also inspect the reference lists of all dientified studies. Studies that have accessible full articles in English will be included.

17 URL to search strategy

If you have one, give the link to your search strategy here. Alternatively you can e-mail this to PROSPERO and we will store and link to it.

I give permission for this file to be made publicly available

No

18 Condition or domain being studied

Give a short description of the disease, condition or healthcare domain being studied. This could include health and wellbeing outcomes.

Abnormal glucose metabolism is causally related to greater risk of several chronic disorders, including diabetes, obesity, dyslipidemia and cardiovascular diseases. Probiotics are one way of altering the gut microbiome and the glucose-lowing effect of probiotics products has raised much interest in recent years.

19 Participants/population

Give summary criteria for the participants or populations being studied by the review. The preferred format includes details of both inclusion and exclusion criteria.

We will include adults (aged >=18, irrespective of age, sex, or race) who are with or without hyperglycemia. Subjects who had undergone intestinal surgery will be excluded.

20 Intervention(s), exposure(s)

Give full and clear descriptions of the nature of the interventions or the exposures to be reviewed

Probiotics are defined as live microorganisms that may have health benefits for the host if consumed in adequate amounts. Probiotic supplemented in any form(e.g. yogurt, capsule, powder, milk). Probiotic supplementation can be in combination with pharmacological (e.g. antidiabetic agents) or non-pharmacological interventions (e.g. diet/lifestyle interventions).

21 Comparator(s)/control

Where relevant, give details of the alternatives against which the main subject/topic of the review will be compared (e.g. another intervention or a non-exposed control group).

Comparison interventions of any type were eligible, e.g. placebo, diet, exercise, pharmacological therapy (e.g. antidiabetic agents).

22 Types of study to be included initially

Give details of the study designs to be included in the review. If there are no restrictions on the types of study design eligible for inclusion, this should be stated.

We will include randomized controlled trials (RCTs). The study design comprised at least a single blind of study participants to probiotics or placebo groups.

23 Context

Give summary details of the setting and other relevant characteristics which help define the inclusion or exclusion criteria.

Studies were included if they met the following inclusion criteria: (1) were human randomized, controlled trials; (2) included adults >=18 years of age with or without hyperglycemia; (3) used probiotic products with live bacteria; (4) the mean fasting blood glucose, along with standard deviation, were reported for the intervention and control groups; (5) subjects had not undergone intestinal surgery; (6) had accessible full articles in English.

24 Primary outcome(s)

Give the most important outcomes.

Fasting blood glucose

Give information on timing and effect measures, as appropriate.

The fasting blood glucose was assessed at the baseline and at end of the intervention period.

25 Secondary outcomes

List any additional outcomes that will be addressed. If there are no secondary outcomes enter None.

Insulin. HOMA-IR

Give information on timing and effect measures, as appropriate.

The outcomes was assessed at the baseline and at end of the intervention period.

26 Data extraction, (selection and coding)

Give the procedure for selecting studies for the review and extracting data, including the number of researchers involved and how discrepancies will be resolved. List the data to be extracted.

Two authors will independently extract the data, check and enter the data into an electronic data collection form. The following information will be abstracted from eligible articles: first author’s last name, publication year and country of origin; study design, including whether parallel or cross-over and single blind or double blind; probiotics, duration of

intervention, sample size, subjects' characteristics including age, sex, body mass index (BMI), baseline blood glucose and antidiabetic medication use will also recorded; probiotics or their fermented dairy products dosage; intervention and treatment results on the levels of blood glucose. If more than one time point for follow-up was reported, we will use the data from the longest follow-up time period. Similarly, we will use the data from the highest dose when there was more than one single dose for the intervention. An electronic data extraction sheet has been developed comprising quality scores and outcome data. Discrepancies will be resolved through discussion.

27 Risk of bias (quality) assessment

State whether and how risk of bias will be assessed, how the quality of individual studies will be assessed, and whether and how this will influence the planned synthesis.

Two review authors will independently assess risk of bias for the included study using the criteria outlined in the Cochrane Handbook for Systematic Reviews of Interventions. We resolved any disagreement by discussion or by involving a third assessor. We will explore the impact of the level of bias through undertaking sensitivity analyses.

28 Strategy for data synthesis

Give the planned general approach to be used, for example whether the data to be used will be aggregate or at the level of individual participants, and whether a quantitative or narrative (descriptive) synthesis is planned. Where appropriate a brief outline of analytic approach should be given.

Meta-analysis of data will be performed using Review Manager software 5.2. The mean difference (mean values±standard deviation) in the Fasting blood glucose, insulin, HOMA-IR for each study were calculated. For parallel trials, mean difference for the outcomes will be calculated as the difference (probiotic diet minus control diet) of the changes (baseline minus endpoint) in mean values. For crossover trials, mean difference for the outcomes will be calculated as the difference (probiotic diet minus control diet) in values at the end of the intervention and control phases. Standard errors and confidence intervals will be converted to standard deviation for the analyses. We will combine data from two period of treatment for cross-over trails. We plan to assess statistical heterogeneity in each meta-analysis using the I-squared statistics. We will regard heterogeneity as substantial if the I-squared is greater than 50% or the I-squared is greater than 25% with a low P value (less than 0.10). We plan to use fixed-effect meta-analysis for combining data where it is reasonable to assume that studies are estimating the same underlying treatment effect. If substantial statistical heterogeneity is detected, we will use random- effects meta-analysis to produce an overall summary, if an average treatment effect across trials is considered clinically meaningful. The random-effects summary will be treated as the average range of possible treatment effects and we will discuss the clinical implications of treatment effects differing between trials. If the average treatment effect is not clinically meaningful, we will not combine trials. If we use random-effects analyses, the results will be presented as the average treatment effect with 95% confidence intervals, and the estimates of Tau-squared and I-squared.

29 Analysis of subgroups or subsets

Give any planned exploration of subgroups or subsets within the review. 'None planned' is a valid response if no subgroup analyses are planned.

If there is a significant heterogeneity among the RCTs, we plan to perform the following subgroup analyses: 1. Past history of hyperglycaemia (yes versus no) 2. Pregnant participants (yes versus no) 3. Probiotic dose (more than 100 billion colony-forming units(CFU) versus less than 100 billion CFU). 4. Probiotic bacterial species (single specie versus more than 1 species). 5. Source of probiotics (capsule versus others) 6. Probiotic duration of treatment (less than 8 weeks versus more than 8 weeks). 7. Use of anti diabetic medications (yes versus no) We will assess subgroup differences by interaction tests available within RevMan. We will report the results of subgroup analyses quoting the I2 statistic and p-value, and the interaction test I2 value. We will perform sensitivity analyses to see whether conclusions are robust to decisions made during the review process following the methods follows: 1. Excluding RCTs with low quality 2. Excluding non-parallel RCTs 3. Excluded heterogeneous studies 4. Studies with double-blind 5. Studies with sample size >20 for each group

Review general information

30 Type of review

Select the type of review from the drop down list.

Intervention

31 Language

Select the language(s) in which the review is being written and will be made available, from the drop down list. Use the control key to select more than one language.

English

Will a summary/abstract be made available in English?
Yes

32 Country
Select the country in which the review is being carried out from the drop down list. For multi-national collaborations select all the countries involved. Use the control key to select more than one country.
China

33 Other registration details
Give the name of any organisation where the systematic review title or protocol is registered together with any unique identification number assigned. If extracted data will be stored and made available through a repository such as the Systematic Review Data Repository (SRDR), details and a link should be included here.

34 Reference and/or URL for published protocol
Give the citation for the published protocol, if there is one.
Give the link to the published protocol, if there is one. This may be to an external site or to a protocol deposited with CRD in pdf format.

I give permission for this file to be made publicly available
Yes

35 Dissemination plans
Give brief details of plans for communicating essential messages from the review to the appropriate audiences.
Do you intend to publish the review on completion?
Yes

36 Keywords
Give words or phrases that best describe the review. (One word per box, create a new box for each term)
Glycaemic control

Glucose

Probiotics

37 Details of any existing review of the same topic by the same authors
Give details of earlier versions of the systematic review if an update of an existing review is being registered, including full bibliographic reference if possible.

38 Current review status
Review status should be updated when the review is completed and when it is published.
Ongoing

39 Any additional information
Provide any further information the review team consider relevant to the registration of the review.

40 Details of final report/publication(s)
This field should be left empty until details of the completed review are available.
Give the full citation for the final report or publication of the systematic review.
Give the URL where available.
