# Supplementary material for: Effect of Probiotics on Glycemic Control: A Systematic Review and Meta-Analysis of Randomized, Controlled Trials
Source: PLoS One. 2015 Jul 10;10(7):e0132121. doi: 10.1371/journal.pone.0132121 (PMC4498615; doi:10.1371/journal.pone.0132121)
Supplement: S3 File — (DOCX) [file pone.0132121.s004.docx]

**Effect of probiotics on glycemic control: a systematic review and meta-analysis of randomized, controlled trials**

**ABSTRACT**

**Background:** Previous clinical trials indicate that probiotic consumption may improve blood glucose control.

**Objective:** We investigated the effects of probiotics on glycemic control with a meta-analysis of randomized, controlled trails.

**Design:** The authors searched in PubMed, Embase, Cochrane Library, and Clinicaltrial.gov for eligible articles up to october 2014. A meta-analysis using a random-effects model was used to analyze combined trails.

**Results:** A meta-analysis of seventeen randomized controlled trials involving 1,105 participants indicated that probiotic consumption, compared with placebo, reduced fasting glucose 0.31 mmol/L (95% CI 0.56, 0.06; p=0.02). The pooled mean difference in insulin was -1.17 μU/mL (95% CI -2.17, -0.41; p=0.004) and the mean difference in homeostasis model assessment-insulin resistant (HOMA-IR) was 0.48 (95% CI -0.83, -0.13; p=0.007). Subgroup analysis suggested better improvement in glucose in hyperglycemic participants than in normoglycemic ones. Better glucose control was obtained with multispecies probiotics compared to single-species probiotics. The duration of intervention ≤ 8 weeks did not significantly reduce glucose. Furthermore, subgroup analysis of trials with a daily dose of probiotic (< 10^11^ colony-forming units, CFUs) did not change glycemic control.

**Conclusions:** Probiotic consumption may improve glycemic control modestly and this effect is greater with a multispecies probiotic used for more than 8 weeks, with a daily dose of ≥ 10^11^ CFUs.

**Introduction**

Abnormal glucose metabolism is causally related to a greater risk of several chronic disorders, including diabetes, obesity, dyslipidemia, and cardiovascular diseases. Blood glucose can be controlled through diet and lifestyle modification to prevent diabetes or related complications and evidence suggests that dietary constituents and supplements such as omega-3 fatty acids [[1](#_ENREF_1)], dairy products [[2](#_ENREF_2)] , pistachio [[3](#_ENREF_3)] and coffee [[4](#_ENREF_4)] can improve glycemic control or reduce an individual’s risk of diabetes.

Probiotics are defined as live microorganisms with potential health benefits for the host if consumed in adequate amounts [[5](#_ENREF_5)]. Probiotic benefits have been investigated for improving immune function [[6](#_ENREF_6)], lowering blood pressure [[7](#_ENREF_7)], and improving lipids [[8](#_ENREF_8)]. Data from animal models suggest that probiotics can reduce blood glucose and insulin resistance [[9](#_ENREF_9)]. Interestingly, research shows that gut microbiota is involved in diabetes and metabolic disorders, revealing that diabetic patients have altered gut microbiota compared to non-diabetic counterparts [[10](#_ENREF_10)]. Probiotics can be used to alter gut microbiota, and its ability to lower glucose is of interest to researchers [[11-13](#_ENREF_11)]. However, human clinical trials of probiotics and glucose have yielded mixed results. For instance, some studies indicate that probiotic yogurt ingestion for 6 weeks can significantly improve glucose [[12](#_ENREF_12)], whereas other studies concluded that this approach had no meaningful effects [[14](#_ENREF_14),[15](#_ENREF_15)]. Such inconsistent findings complicate approaches to and conclusions about probiotic use. Therefore, we systematically examined the effect of probiotics on glucose control using a meta-analysis of randomized controlled trials (RCTs).

**Materials and Methods**

**1. Literature search**

The online databases PubMed, The Cochrane Library, EMBASE, and Clinicaltrial.gov were searched until October 2014 for relevant studies. The following terms were used to search for relevant publication: ‘probiotic’, ‘lactobacilli’, ‘bifidobacter’, ‘bacillus’, ‘saccharomyces’, ‘enterococcus’, ‘streptococcus’, ‘yogurt’, ‘yoghurt’, ‘sour milk’, ‘fermented milk’, ‘gut microbiota’ in combination with ‘glucose’, ‘blood sugar’, ‘glycemic’, ‘hyperglycemia’. We supplemented the literature search by scanning the reference lists of relevant articles. The methodology of this systematic review were specified in advance and documented in a protocol that was published in a prospective register of systematic reviews, PROSPERO (www.crd.york.ac.uk/PROSPERO; ref CRD42014014498).

**2. Study selection**

Studies were included if they meet the following criteria: (1) human RCTs, (2) included adults ≥ 18 years-of-age with or without hyperglycemia, (3) use of probiotic products as an intervention group, (4) mean fasting blood glucose (+ SD) were reported for the intervention and control groups, (5) subjects had not received intestinal surgery, (6) studies with high methodology equality (through assessment of studies equalities, the risk of bias figure was in Fig. S1). Studies were excluded if the total number of probiotic bacteria was not reported, if the probiotic contained prebiotics as the intervention product, or were not in English.

YTR and JH conducted an initial screening of studies based on titles and then reviewed abstracts and the full text to assess eligibility criteria independently. Final eligibility was determined through agreement between the 2 reviewers, with any disagreement resolved in consultation with HC. The PRISMA flow chart summarizes these decisions (Fig. 1). Included articles were analyzed for publication bias and relevant data were extracted.

**Fig. 1. Flow diagram of studies evaluated in the systematic review.**

**4. Data extraction**

YTR and JH independently extracted these data from eligible publications: probiotics, duration of intervention, sample size, subjects’ characteristics including age, sex, body mass index (BMI), baseline blood glucose, and antidiabetic medication use; probiotics or their fermented dairy products dosage; intervention and treatment results on glucose. We also note data on baseline and follow-up insulin concentrations and HOMA-IR to measure any correlation between probiotics and glycemic control.

**5. Data analyses**

Probiotic use on glycemic control is defined as the mean difference in glucose changes between the intervention and control groups. Statistical analysis was performed according to the *Cochrane Handbook for Statistical Review of Interventions (Version 5.0.2)*. Mean glucose differences were pooled using a s-effects model due to study heterogeneity.

Heterogeneity was tested and measured with a Q-test and with I^2^ statistics. In general, we regarded heterogeneity as substantial if the I^2^ > 50% or I^2^ > 25% with a low p value (< 0.10). We explored sources of heterogeneity by comparing mean differences in glucose between subgroups stratified by hyperglycemia, pregnancy, probiotic dose, species, and sources and duration of treatment. To test data robustness, sensitivity analyses were used for excluding small studies (n <20 sample), and data were reanalyzed using a fix-effects model after excluding studies of high heterogeneity that were limited to a double-blind trail.

Potential publication bias was assessed using funnel plots. If publication bias was detected, a sensitivity analysis of smaller studies reporting more extreme effect sizes were excluded. The meta-analysis was performed using RevMan software (Cochrane Review Manager, version 5.2). Statistical tests were two-sided (p < 0.05).

**Results**

**1. Characteristics of Included Studies**

Seventeen clinical trials involving 1,105 participants (551 probiotics, 554 control) were included and these trials were parallel RCTs similar with regards to baseline characteristics, indicating successful randomization. Fifteen studies used a double-blind design [[12](#_ENREF_12),[14-27](#_ENREF_14)]; one was a single-blind design [[28](#_ENREF_28)]. Fourteen studies reported similarity in intervention and placebo [[12](#_ENREF_12),[14-16](#_ENREF_14),[18-27](#_ENREF_18)] and eight studies included blinding of treatment allocation and measurements [[12](#_ENREF_12),[14](#_ENREF_14),[18](#_ENREF_18),[20](#_ENREF_20),[21](#_ENREF_21),[23](#_ENREF_23),[24](#_ENREF_24),[26](#_ENREF_26)]. In eleven trails, dropout reasons and numbers were noted [[12](#_ENREF_12),[14](#_ENREF_14),[16](#_ENREF_16),[18-21](#_ENREF_18),[25-28](#_ENREF_25)]. Funnel plots of studies were slightly asymmetrical, which may be interpreted as publication bias (Fig. S2).

Study characteristics are presented in Table 1. The mean baseline fasting blood glucose (FBG) across the studies was 5.89 mmol/L in probiotic group and 5.83 mmol/L in the control group. In five trails, patients used antidiabetic medications but they did not change their medications during the study [[12](#_ENREF_12),[16](#_ENREF_16),[22](#_ENREF_22),[25](#_ENREF_25),[27](#_ENREF_27)]. The duration of the studies ranged from 3 to 24 weeks and nutrition intake was measured in 7 studies [[12](#_ENREF_12),[16](#_ENREF_16),[20-22](#_ENREF_20),[25](#_ENREF_25),[28](#_ENREF_28)], and no differences in energy or nutrient intake between intervention and control groups was found. The remainder of the studies only reported that participants were advised to maintain their diet, except for 2 studies in which subjects were instructed to modify dietary intake in both groups [[20](#_ENREF_20),[26](#_ENREF_26)]. Probiotic species and dose used varied between studies. Eight studies used a single species of probiotics, whereas the others used a combination of more than 2 species. All studies reported good compliance with no side effects of consuming probiotic, except 2 studies that reported subject flatulence, loose stools or constipation [[18](#_ENREF_18),[20](#_ENREF_20)].


One study reported results in two subsets. Ivey and coworkers [[14](#_ENREF_14)] compared the effect of probiotic in four arms (1 probiotic capsule group; 1 placebo capsule group; 1 probiotic yoghurt group and 1 control milk group ). We analyzed subsets separately and 16 articles and 17 RCTs were included in this meta-analysis.

**2. Main outcomes**

All studies reported changes in fasting blood glucose (FBG). Of the seventeen trails, four studies reported a significant reduction of FBG after probiotic intervention, with mean differences ranging from -0.15 to -1.51 mmol/L [[12](#_ENREF_12),[16](#_ENREF_16),[20](#_ENREF_20),[24](#_ENREF_24)]. The meta-analysis of 17 trails showed a significant reduction of FBG of 0.31 mmol/L (95% CI 0.56, 0.06; p=0.02) compared with control groups. More heterogeneity was observed across studies (*I*^2^ = 92%, p < 0.01) (Fig. 2).

Eleven studies also reported changes in insulin, with 3 studies reporting a significant reduction of insulin after probiotic use [[23](#_ENREF_23),[24](#_ENREF_24),[28](#_ENREF_28)]. The mean difference ranged from -0.36 to -3.8 μU/mL. The pooled mean difference was -1.29 μU/mL (95% CI -2.17, -0.41; p = 0.004) for insulin (Fig. 3A). Eight of 17 studies reported changes in HOMA-IR, with 4 studies reporting a significant reduction of HOMA-IR after consuming probiotics[[16](#_ENREF_16),[23](#_ENREF_23),[24](#_ENREF_24),[28](#_ENREF_28)]. The mean difference ranged from -0.41 to -1.60. The pooled mean difference was -0.48 (95% CI -0.83, -0.13; p = 0.007) for HOMA-IR (Fig. 3B).

**3. Subgroup and sensitivity analyses**

Subgroup analysis of studies with hyperglycemic patients revealed a significant reduction of (1.46 mmol/L) FBG, and these results were not reported in normoglycemic patients. However, limiting participants to those using antidiabetic medications increased the improvements in FBG compared with subjects not using medications (-0.98 vs. -0.14 mmol/L). Limiting participants to pregnant women did not change results between probiotics consumed and improvement in glycemic control. Reduced glucose in trials that include multispecies probiotics was more pronounced than use of a single species of probiotics. Duration of intervention > 8 weeks resulted in a significant reduction in glucose. However, limiting the analysis to those interventions with duration of intervention ≤ 8 weeks did not offer the same results. Use of probiotic capsules significantly reduced FBG, and similar results were not found for other probiotic sources. A daily dose of probiotics ≥ 10^11^ CFU had better glucose-reducing effects compared with those studies using < 10^11^ CFU (-0.62 vs. -0.28 mmol/L) (Table 2).


Sensitivity analysis of individual studies showed that high heterogeneity was influenced by 4 trails [[14](#_ENREF_14),[16](#_ENREF_16),[24](#_ENREF_24)]. Excluding these studies and using fixed-effects model revealed a similarity with the pooled mean difference in glucose (p < 0.01). Excluding studies with small sample sizes (n < 20) [[15](#_ENREF_15),[17](#_ENREF_17),[23](#_ENREF_23),[24](#_ENREF_24),[26](#_ENREF_26)], we found a significant reduction in glucose (p = 0.04). Limiting the analysis to double-blind trails revealed a significant reduction in glucose as well. Sensitivity analysis also indicated a similarity with the overall pooled mean difference in insulin and HOMA-IR (Table 3).


**Discussion**

This is the first study to systematically analyze the effect of probiotics on glycemic control. Overall, probiotics significantly reduced FBG by 0.31 mmol/L and insulin by 1.17 μU/mL and improved HOMA-IR by 0.48, indicating a modest effect of probiotics on glycemic control; however, even small glucose reductions may offer health benefits. Abnormal glucose metabolism carries significant risks for many metabolic diseases, such as obesity, diabetes, obstructive sleep apnea-hypopnea syndrome, and cardiovascular disease.

The hypothesis that probiotics may be involved in maintenance of healthy gut microbiota and glucose management has received much attention. The ratio of bacteroidete species in T2DM correlates positively with plasma glucose [[29](#_ENREF_29)] and alterations in gut microbiota have recently been reported in patients with T2DM, and this may be reversible with probiotic supplement [[10](#_ENREF_10)]. Dietary supplementation of probiotics for high fructose- and streptozotocin-induced diabetes in rats improved glucose and lipid metabolism, suppressed glucose intolerance and delayed the onset of hyperglycemia, hyperinsulinemia, dyslipidemia, and oxidative stress [[30](#_ENREF_30),[31](#_ENREF_31)]*.* Yun’s group [[32](#_ENREF_32)] found that FBG and 2 h blood glucose were significantly lower after probiotic ingestion for 3 weeks in db/db mice. We observed that probiotics had a slightly greater effect on FBG in hyperglycemic participants. However, limiting participants to those who did not use oral hypoglycemic agents yielded nonsignificant effects on FBG, indicating that blood glucose alterations may not be solely due to probiotic products. Antidiabetic medications might offset the glucose-lowing effect of probiotics, even though all studies reported no change in the use of glucose-lowing medication.

Administration of probiotic sources varied among trails with most trials using encapsulated probiotic supplements. Subgroup analysis of studies using the probiotic capsule revealed significant reductions in FBG compared with other sources. However, an inadequate number of studies that used other sources of probiotics (yogurt, rose-hip drinks, probiotic cheese, et al) limits these conclusions for the best source of probiotics. Moreover, not all studies reported beneficial effects of probiotics, and thus caution should be taken in terms of the species and dosage to be used, which may have important ramifications on the effects observed. Subgroup analysis of studies using multispecies of probiotic indicated a more pronounced reduction in FBG compared with those using a single species of probiotic. The findings of present meta-analysis are in line with the previous studies, both suggesting a combination of probiotic species are more effective than single species products [[33](#_ENREF_33)]. In addition, subgroup analysis indicated that the reduction in FBG was greater when the daily dose of probiotics consumption ≥ 10^11^ CFU. Although this finding may provide important information for future study, so caution is required — positive effects may be due to the low numbers of RCTs included in the subgroup analysis.

Another important observation we made was that longer treatments (> 8 weeks) with probiotics affected FBG more than short treatments. However, further studies with different treatment durations are required to confirm this result. In addition, pregnant women are susceptible to increased insulin resistance and glucose, so a subgroup analysis was conducted to limit data to pregnant women. In human clinical trials, supplementation of probiotics combined with dietary counseling has been shown to positively affect glucose metabolism in normoglycemic pregnant women [[20](#_ENREF_20)]*.* However, the subgroup analysis of probiotics and FBG was not significant among pregnant women, which might be explained by the inter-individual differences of pregnancy. Furthermore, probiotic strain differences, dose, and treatment duration across different studies might explain differences in outcomes.

How probiotics lower glucose is unclear. They may be related to decreased oxidative stress [[12](#_ENREF_12)] which his shown to be present in hyperglycemia [[34](#_ENREF_34)]. Specific strains of lactic acid bacteria have antioxidant properties [[35](#_ENREF_35),[36](#_ENREF_36)]. For example, Yadav and colleagues [[30](#_ENREF_30)] reported that probiotic dahi, a fermented milk containing *Lactobacillus acidophilus* and *L. casei* delayed the progression of glucose intolerance, hyperglycemia, hyperinsulinemia via decreased oxidative stress in animal models. Also, low-grade chronic inflammation is observed in diabetic and obese individuals and the immune system is crucial for regulation of glucose metabolism. Thus, probiotics may modulate immune responses and systemic low-grade inflammation, in particular by reducing cytokines [[37](#_ENREF_37)] and suppressing the NF-κB pathway, which mediates immune system microbial activation via toll-like receptors [[38](#_ENREF_38)]. Laitinen’s group [[20](#_ENREF_20)] observed pronounced effects of probiotics on reduced glucose and attributed this to immunoregulatory properties. Five studies suggest that the consumption of probiotics decreased inflammatory markers, including hsCRP, IL-6, and TNF-α [[15](#_ENREF_15),[16](#_ENREF_16),[22-24](#_ENREF_22)]. Also, studies indicate that systemic inflammation was reduced and intestinal endotoxin (a potential inflammatory stimulant) was decreased with probiotics, lowering insulin resistance and hyperglycemic incidences [[39](#_ENREF_39)] Probiotics may attenuate circulating endotoxin, subsequently affecting glucose metabolism [[40](#_ENREF_40),[41](#_ENREF_41)]

Our meta-analysis revealed a prominent beneficial effect of probiotics on glycemic control along with lower insulin and HOMA-IR, data that are consistent with a recent meta-analysis suggesting that yogurt intake was associated with an 18% lower risk of T2DM[[42](#_ENREF_42)] .

Our work has several limitations. First, we could not obtain data from unpublished literature or non-English published material, which may increase publication bias. Second, some studies had fewer than 20 participants for each experimental group. Funnel plots show possible bias, favoring small trails with extreme effects. However, these trails had small weights in our meta-analysis and excluding them only slightly modified probiotic-induced effects on glucose. Third, two studies were relatively short (3 to 4 weeks of probiotic consumption[21,[26](#_ENREF_26)]) and such brief studies may affect the meta-analysis data because subgroup analysis of studies shorter than 8 weeks did not show significant reductions in fasting blood glucose. Therefore, more RCTs with larger samples and longer study durations are needed to conclude that probiotics of different species and doses has an effect on glycemic control.

Thus, consumption of probiotics may improve fasting blood glucose and reduce insulin and HOMA-IR. Modification of gut microbiota by probiotic supplementation may be a method for preventing and control hyperglycemia in clinical practice.

**Figure Legends**

**Fig. 1. Flow diagram of studies evaluated in the systematic review.**

**Fig. 2. Forest plot of randomized controlled trials in adults comparing probiotics with placebo/comparator.** Weighted mean differences (95% CIs) for fasting blood glucose are shown. Pooled estimates (*diamonds*) calculated by the random effects method. IV, inverse variance.

**Fig. 3. Forest plot of RCTs comparing probiotics with placebo/comparators.** Weighted mean differences (95% CIs) for insulin (A) and HOMA-IR (B) are shown. Pooled estimates (*diamonds*) calculated by the random effects method. IV, inverse variance.

**Figures**

**Figure 1.**


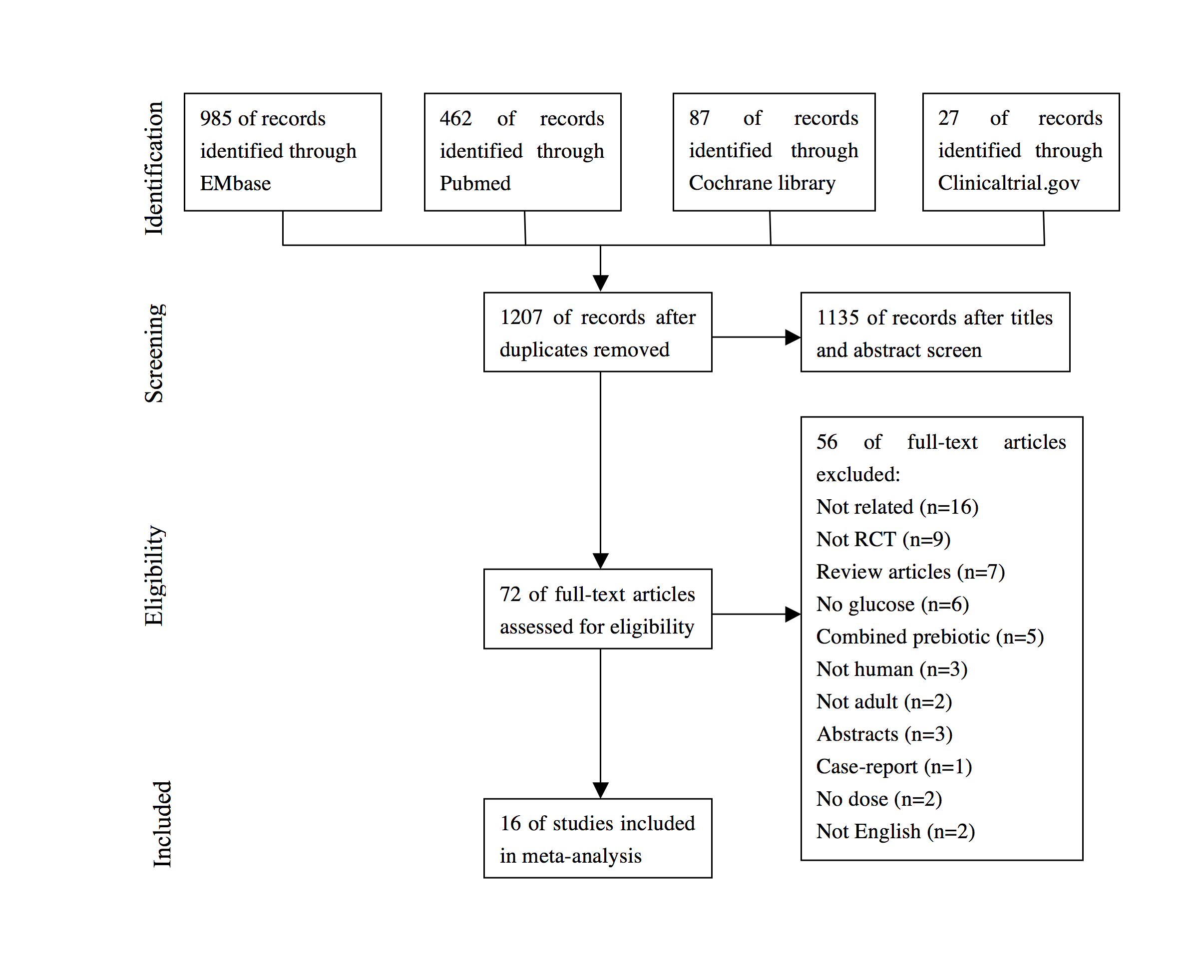


**Figure 2.**


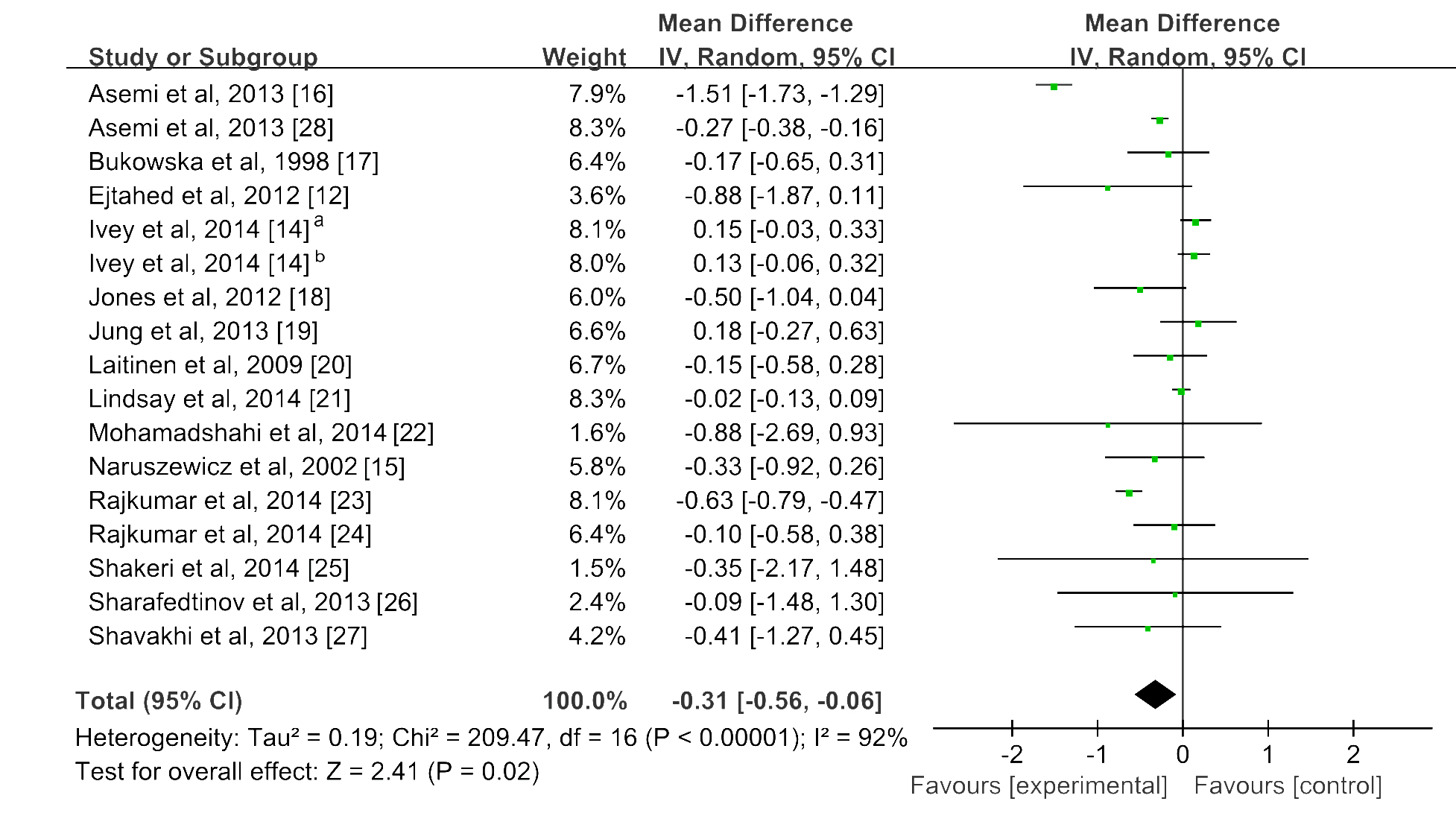


**Figure 3**


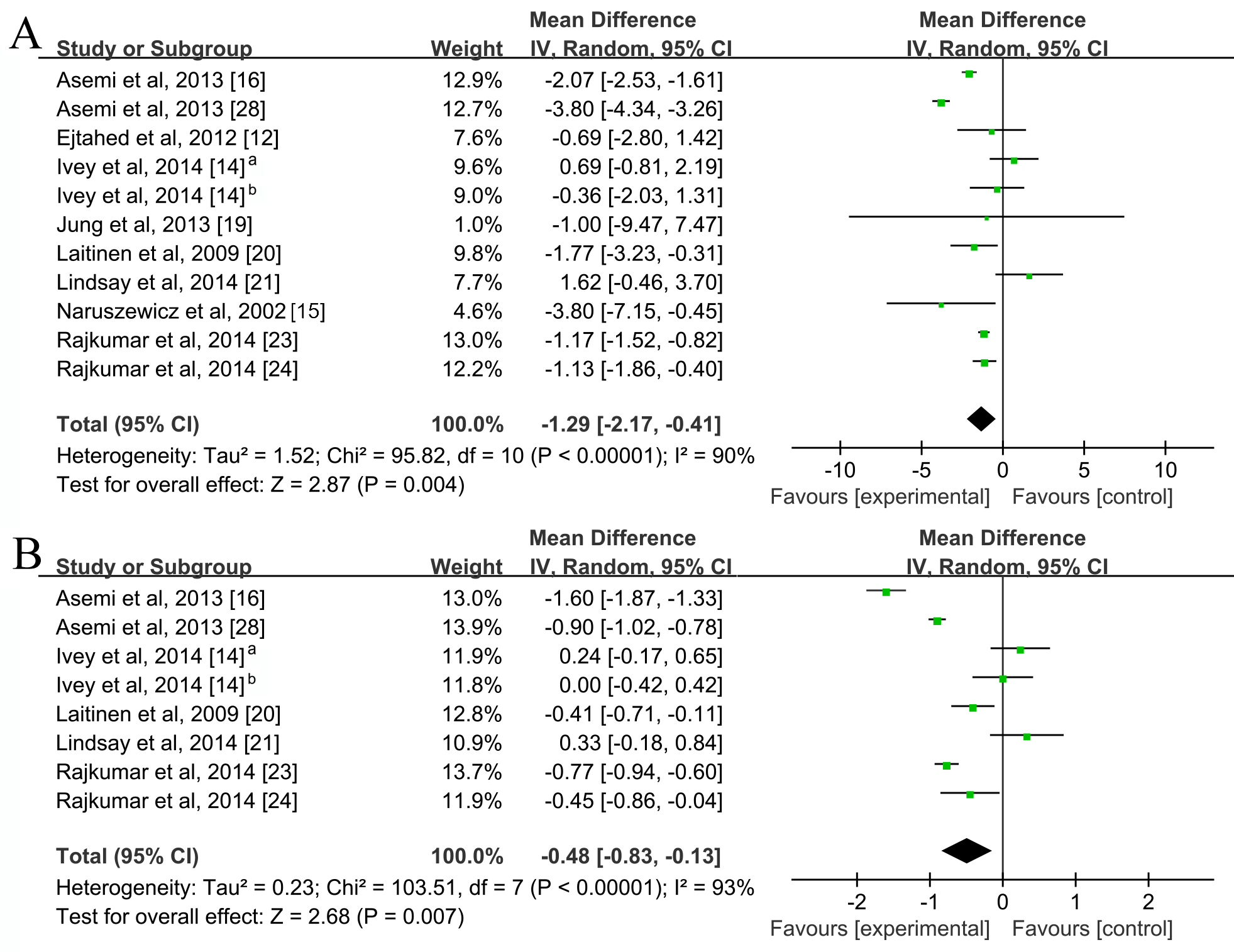


**Tables**

**Table 1. Characteristics of included studies**

| \| **Study** \| **Design, Location** \| **Probiotic Source** \| **Duration (weeks)** \| **Participant, Age (No. of Intervention/No. of Control)** \| **Baseline Characteristics** \| \| \| \| **Probiotic** \| **Dose, CFU** \| **Antidiabetic Medication Use** \| \| --- \| --- \| --- \| --- \| --- \| --- \| --- \| --- \| --- \| --- \| --- \| --- \| \| *Glucose (mmol/l)* \| *BMI (kg/㎡)* \| *Insulin (μU/ml)* \| *HOMA-IR* \| \| Asemi *et al.* (16) \| DB,PC,P, Iran \| C \| 8 \| T2DM, 35-70, (27/27) \| 7.73 \| 30.89 \| 5.76 \| 2.01 \| *L. acidophilus, L. rhamnosus, L. casei, L. bulgaricus, B. longum, S. thermophilus* \| 3.92×10^10^ \| YES \| \| Asemi *et al.* (28) \| SB,PC,P, Iran \| Y \| 9 \| Pregnant, 18-30, (37/33) \| 5.21 \| ND \| 7.90 \| 1.82 \| *S. thermophilus, L. bulgaricus, L. acidophilus, B. animalis* \| 1×10^7^ \| NO \| \| Bukowska *et al.* (17) \| DB,PC,P, Poland \| Fermented oatmeal soups \| 6 \| HC, men, 40-45（15/15） \| 5.94 \| 26.25 \| ND \| ND \| *L. plantarum* \| 1×10^10^ \| NO \| \| Ejtahed *et al.* (12) \| DB,PC,P, Iran \| Y \| 6 \| T2DM,30-60, (30/30) \| 7.71 \| 29.05 \| 6.89 \| ND \| *L. acidophilus, B. lactis* \| 3.98×10^9^ \| YES \| \| Ivey *et al.* (14)^a^ \| DB,PC,P, Australia \| C, Y \| 6 \| OB, 56-77, (40/37） \| 5.58 \| 30.41 \| 9.79 \| 2.47 \| *L. acidophilus, B. animalis subsp lactis* \| 6×10^9^ \| NO \| \| Ivey *et al.* (14)^b^ \| DB,PC,P, Australia \| C \| 6 \| OB, 56-77, (40/39） \| 5.47 \| 30.80 \| 9.88 \| 2.44 \| *L. acidophilus, B. animalis subsp lactis* \| 3×10^9^ \| NO \| \| Jones *et al.* (18) \| DB,PC,P, Canada \| C \| 9 \| HC,20-75, (67/64） \| 5.35 \| 27.30 \| ND \| ND \| *L. reuteri* \| 5.8×10^9^ \| NO \| \| Jung *et al.* (19) \| DB,PC,P, Korea \| C \| 6 \| OB,19-60, (22/28） \| 5.75 \| 29.16 \| 10.21 \| ND \| *L. Gasseri* \| 6×10^10^ \| NO \| \| Laitinen *et al.* (20) \| DB,PC,P, Finland \| C \| 20 \| Pregnant,25-35, (66/70） \| 4.53 \| ND \| 5.67 \| 1.17 \| *L. rhamnosus, B. lactis* \| 1×10^10^ \| NO \| \| Lindsay *et al.* (21) \| DB,PC,P, Ireland \| C \| 4 \| OB, pregnant, 31-36, (63/75) \| 4.73 \| 33.55 \| 15.36 \| 3.27 \| *L. salivarius* \| 1×10^9^ \| NO \| \| Mohamadshahi al (22) \| DB,PC,P，Iran \| Y \| 8 \| OB,T2DM, 42-59, (20/20) \| 10.07 \| 28.79 \| ND \| ND \| *L. Bb12, L. acidophilus* \| 1.11×10^9^ \| ND \| \| Naruszewicz *et al.* (15) \| DB,PC,P, Sweden \| D \| 6 \| Healthy smoker, 35-45,（18/18） \| 5.89 \| 25.3 \| 9.7 \| ND \| *L. plantarum* \| 2×10^10^ \| NO \| \| Rajkumar *et al.* (23) \| DB,PC,P, India \| C \| 6 \| OB, 40-60,（15/15) \| 4.93 \| 28.79 \| 18.15 \| 3.95 \| *L. acidophilus, L. paracasei, L. delbrueckii, L. plantarum, B. longum, B. infantis, B. breve* \| 1.13×10^11^ \| NO \| \| Rajkumar *et al.* (24) \| DB,PC,P, Japan \| C \| 6 \| Health, 20-25, (15/15) \| 4.70 \| 22.53 \| 18.77 \| 3.80 \| *L. salivarius* \| 4×10^9^ \| NO \| \| Shavakhi *et al.* (25) \| DB,PC,P, Iran \| C \| 24 \| NASH, 18-75, (31/32) \| 5.52 \| 28.40 \| ND \| ND \| *L. acidophilus, L. rhamnosus L. casei, L. bulgaricus, L. rhamnosus, L. bulgaricus* \| 9.5×10^8^ \| YES \| \| Shakeri *et al.* (26) \| DB,PC,P, Iran \| Bread \| 8 \| T2DM, 35-70, (26/26) \| 8.27 \| 30.05 \| ND \| ND \| *L. sporogenes* \| 1.30×10^10^ \| YES \| \| Sharafedtinov *et al.* (27) \| DB,PC,P, Estonia \| Cheese \| 3 \| Met,S, 30-69, (25/11) \| 7.06 \| 37.27 \| ND \| ND \| *L. plantarum* \| 7.5×10^12^ \| NO \| |
| --- | --- | --- | --- | --- | --- | --- | --- | --- | --- | --- | --- | --- | --- | --- | --- | --- | --- | --- | --- | --- | --- | --- | --- | --- | --- | --- | --- | --- | --- | --- | --- | --- | --- | --- | --- | --- | --- | --- | --- | --- | --- | --- | --- | --- | --- | --- | --- | --- | --- | --- | --- | --- | --- | --- | --- | --- | --- | --- | --- | --- | --- | --- | --- | --- | --- | --- | --- | --- | --- | --- | --- | --- | --- | --- | --- | --- | --- | --- | --- | --- | --- | --- | --- | --- | --- | --- | --- | --- | --- | --- | --- | --- | --- | --- | --- | --- | --- | --- | --- | --- | --- | --- | --- | --- | --- | --- | --- | --- | --- | --- | --- | --- | --- | --- | --- | --- | --- | --- | --- | --- | --- | --- | --- | --- | --- | --- | --- | --- | --- | --- | --- | --- | --- | --- | --- | --- | --- | --- | --- | --- | --- | --- | --- | --- | --- | --- | --- | --- | --- | --- | --- | --- | --- | --- | --- | --- | --- | --- | --- | --- | --- | --- | --- | --- | --- | --- | --- | --- | --- | --- | --- | --- | --- | --- | --- | --- | --- | --- | --- | --- | --- | --- | --- | --- | --- | --- | --- | --- | --- | --- | --- | --- | --- | --- | --- | --- | --- | --- | --- | --- | --- | --- | --- | --- | --- | --- | --- | --- | --- | --- | --- | --- | --- | --- | --- | --- | --- | --- | --- | --- |

C: capsule; Y: yogurt; D: drink; CFU, colony-forming unit; DB, double blind; HC, hypercholesterolemia; Met.S, metabolic syndrome; NASH, non-alcoholic steatohepatitis; ND, not detected; OB, obesity; P, parallel; PC, placebo control; SB, single blind; T2DM, type 2 diabetes mellitus.

**Table 2. Subgroup analysis of included RCTs**

|  | ***Blood Glucose (mmol/L)*** | | | | | | |
| --- | --- | --- | --- | --- | --- | --- | --- |
| ***Groups*** | ***Trials*** | ***Weight mean difference*** | | ***95% CI*** | ***P*** | ***I^2^ (%)*** | ***P _heterogeneity_*** |
| Hyperglycemic | | | | | | | |
| YES | 4 | | -1.46 | -1.67, -1.25 | <0.01 | 11 | 0.34 |
| NO | 13 | | -0.15 | -0.33, 0.02 | 0.09 | 82 | <0.01 |
| Pregnant participant | | | | | | | |
| YES | 3 | | -0.15 | -0.35, 0.05 | 0.16 | 81 | <0.01 |
| NO | 14 | | -0.36 | -0.74, 0.01 | 0.06 | 93 | <0.01 |
| Duration | | | | | | | |
| ＞8 weeks | 4 | | -0.27 | -0.37, -0.17 | <0.01 | 0 | 0.78 |
| ≤8 weeks | 13 | | -0.32 | -0.67, 0.02 | 0.06 | 94 | <0.01 |
| Source of probiotic | | | | | | | |
| Capsule | 8 | | -0.24 | -0.45, 0.02 | 0.03 | 84 | <0.01 |
| Others | 9 | | -0.42 | -1.01, 0.18 | 0.17 | 95 | <0.01 |
| Species |  | |  |  |  |  |  |
| Single species | 8 | | -0.05 | -0.14, 0.05 | 0.36 | 0 | 0.64 |
| Multispecies | 9 | | -0.44 | -0.83, -0.05 | 0.03 | 96 | <0.01 |
| Daily dose | | | | | | | |
| ≥1,011 CFU | 2 | | -0.62 | -0.78，-0.47 | <0.01 | 0 | 0.45 |
| <1,011 CFU | 15 | | -0.28 | -0.56, -0.01 | 0.04 | 92 | <0.01 |
| Use of antidiabetic medications | | | | | | | |
| YES | 5 | | -0.98 | -1.58,-0.37 | <0.01 | 54 | 0.07 |
| NO | 12 | | -0.14 | -0.32, 0.04 | 0.12 | 82 | <0.01 |
| Total | 17 | | -0.31 | -0.56, -0.05 | 0.02 | 92 | <0.01 |

Data were meta-analyzed by using a random-effects model or fixed-effects model as appropriate and are presented as WMD. Statistical heterogeneity was assessed by using he chi-square test and quantified by using the I^2^ statistic.

**Table 3. Sensitivity analysis of RCTs**

| ***Groups*** | ***Trials*** | | ***WMD*** | ***95% CI*** | ***P*** | | | ***I^2^ (%)*** | | | ***P heterogeneity*** | |
| --- | --- | --- | --- | --- | --- | --- | --- | --- | --- | --- | --- | --- |
| Blood Glucose (mmol/L) | | | | | | | | | | | | |
| Excluded heterogeneous studies | 13 | | -0.16 | -0.23, -0.09 | | <0.01 | | 31 | | | 0.13 | |
| Studies with double-blind | 16 | | -0.31 | -0.62, -0.01 | | 0.04 | | 93 | | | <0.01 | |
| Studies with sample size ≥ 20 | 9 | | -0.18 | -0.35, -0.01 | | 0.04 | | 53 | | | 0.03 | |
| Insulin (μU/ml) | | | | | | | | | | | | |
| Excluded heterogeneous studies | 7 | -1.17 | | -1.48, -0.87 | | | <0.01 | | | 0 | | 0.66 |
| HOMA-IR | | | | | | | | | | | | |
| Excluded heterogeneous studies | 3 | -0.31 | | -0.57, -0.05 | | | 0.02 | | 32 | | 0.22 | |

WMD, weight mean difference.
