# Supplementary material for: Effect of Probiotics on Glycemic Control: A Systematic Review and Meta-Analysis of Randomized, Controlled Trials
Source: PLoS One. 2015 Jul 10;10(7):e0132121. doi: 10.1371/journal.pone.0132121 (PMC4498615; doi:10.1371/journal.pone.0132121)
Supplement: S4 File — (PDF) [file pone.0132121.s005.pdf]

CERTIFICATE OF ENGLISH EDITING

Manuscript Title:

Effect of probiotics on glycemic control: a systematic review and meta-analysis of randomized, controlled trials

Date Issued:

March 3, 2015

This document certifies that the manuscript listed above was copy edited for proper English language at Accdon. LetPub is the flagship professional editing brand of Accdon. All of our language editors are native English speakers with long-term experience in editing scientific and technical manuscripts. They are committed to leveling the playing field for researchers whose native language is not English.

- Neither the research content nor the authors' intended meaning were altered in any way during the editing process.
- Documents receiving this certification should be considered ready for publication where language issues are concerned. However, the authors may accept or reject Accdon's suggestions and changes at their own discretion.
- If you have any questions or concerns about this edited document, please contact Accdon at support@letpub.com

LetPub
Your Manuscript Editor

Accdon specializes in editing, translation, proofreading, and publication counseling, working with researchers and publishers around the world. For more information about our company, services, and partner discounts, please visit www.letpub.com.
