# Supplementary material for: Effect of Probiotics on Glycemic Control: A Systematic Review and Meta-Analysis of Randomized, Controlled Trials
Source: PLoS One. 2015 Jul 10;10(7):e0132121. doi: 10.1371/journal.pone.0132121 (PMC4498615; doi:10.1371/journal.pone.0132121)
Supplement: S5 File — (DOCX) [file pone.0132121.s006.docx]

**Reference list of excluded articles after full text assessment**

**No glucose: the data is not convertible or presented (n=12):**

1. Rizkalla SW, Luo J, Kabir M, Chevalier A, Pacher N, et al. (2000) Chronic consumption of fresh but not heated yogurt improves breath-hydrogen status and short-chain fatty acid profiles: A controlled study in healthy men with or without lactose maldigestion. American Journal of Clinical Nutrition 72: 1474-1479. **(Cannot extract the SD of glucose in each group)**

2.Barreto FM, Colado SAN, Morimoto HK, Batisti LMA, Dichi I, et al. (2014) Beneficial effects of Lactobacillus plantarum on glycemia and homocysteine levels in postmenopausal women with metabolic syndrome. Nutrition 30: 939-942. **(Can not extract the SD of glucose in each group)**

3. Alisi A, Bedogni G, Baviera G, Giorgio V, Porro E, et al. (2014) Randomised clinical trial: The beneficial effects of VSL#3 in obese children with non-alcoholic steatohepatitis. Aliment Pharmacol Ther 39: 1276-1285. **(Can not extract the SD of glucose in each group)**

4.Hong YS, Hong KS, Park MH, Ahn YT, Lee JH, et al. (2011) Metabonomic understanding of probiotic effects in humans with irritable bowel syndrome. Journal of Clinical Gastroenterology 45: 415-425. **(The glucose data was measured by other methods and cannot be normalized)**

5. Dong H, Rowland I, Thomas LV, Yaqoob P (2013) Immunomodulatory effects of a probiotic drink containing Lactobacillus casei Shirota in healthy older volunteers. Eur J Nutr 52: 1853-1863. **(The glucose data was not presented and we tried to get it from the author but failed)**

6. Andreasen AS, Larsen N, Pedersen-Skovsgaard T, Berg RM, Moller K, et al. (2010) Effects of Lactobacillus acidophilus NCFM on insulin sensitivity and the systemic inflammatory response in human subjects. Br J Nutr 104: 1831-1838. **（Only pre-intervention data of glucose data can be extracted）**

7. Okazaki M, Matsukuma S, Suto R, Miyazaki K, Hidaka M, et al. (2013) Perioperative synbiotic therapy in elderly patients undergoing gastroenterological surgery: a prospective, randomized control trial. Nutrition 29: 1224-1230. (**The study outcome did not mention blood glucose)**

8. Kadooka Y, Sato M, Imaizumi K, Ogawa A, Ikuyama K, et al. (2010) Regulation of abdominal adiposity by probiotics (Lactobacillus gasseri SBT2055) in adults with obese tendencies in a randomized controlled trial. Eur J Clin Nutr 64: 636-643. (**The study outcome did not mention blood glucose)**

9. Luoto R, Laitinen K, Nermes M, Isolauri E (2010) Impact of maternal probiotic-supplemented dietary counselling on pregnancy outcome and prenatal and postnatal growth: a double-blind, placebo-controlled study. Br J Nutr 103: 1792-1799. (**The study outcome did not mention blood glucose)**

10. Mohamadshahi M, Veissi M, Haidari F, Javid AZ, Mohammadi F, et al. (2014) Effects of probiotic yogurt consumption on lipid profile in type 2 diabetic patients: A randomized controlled clinical trial. J Res Med Sci 19: 531-536. (**The study outcome did not mention blood glucose)**

11. Vajro P, Mandato C, Licenziati MR, Franzese A, Vitale DF, et al. (2011) Effects of Lactobacillus rhamnosus strain GG in pediatric obesity-related liver disease. J Pediatr Gastroenterol Nutr 52: 740-743. (**The study outcome did not mention blood glucose)**

12.Luoto R, Laitinen K, Nermes M, Isolauri E (2012) Impact of maternal probiotic-supplemented dietary counseling during pregnancy on colostrum adiponectin concentration: a prospective, randomized, placebo-controlled study. Early Hum Dev 88: 339-344. (**The study outcome did not mention blood glucose)**

**Review articles or abstracts (10):**

1.Sanders ME, Klaenhammer TR, Ouwehand AC, Pot B, Johansen E, et al. (2014) Effects of genetic, processing, or product formulation changes on efficacy and safety of probiotics. Ann N Y Acad Sci 1309: 1-18.

2.Messaoudi S, Manai M, Kergourlay G, Prevost H, Connil N, et al. (2013) Lactobacillus salivarius: bacteriocin and probiotic activity. Food Microbiol 36: 296-304.

3.Lye HS, Kuan CY, Ewe JA, Fung WY, Liong MT (2009) The improvement of hypertension by probiotics: Effects on cholesterol, diabetes, renin, and phytoestrogens. International Journal of Molecular Sciences 10: 3755-3775.

4.Panwar H, Rashmi HM, Batish VK, Grover S (2013) Probiotics as potential biotherapeutics in the management of type 2 diabetes - prospects and perspectives. Diabetes/Metabolism Research and Reviews 29: 103-112.

5.Saad SMI (2006) Probiotics and prebiotics: The state of the art. Revista Brasileira de Ciencias Farmaceuticas/Brazilian Journal of Pharmaceutical Sciences 42: 1-16.

6.Fukuda S, Ohno H (2014) Gut microbiome and metabolic diseases. Seminars in Immunopathology 36: 103-114.

7.De Bandt JP, Waligora-Dupriet AJ, Butel MJ (2011) Intestinal microbiota in inflammation and insulin resistance: Relevance to humans. Current Opinion in Clinical Nutrition and Metabolic Care 14: 334-340.

8. Nayak S (2014) Role of probiotics in maternal nutrition. Perinatology 15: 62-64.

9. Brahe L, le Chatelier E, Prifti E, Kennedy S, Blaedel T, et al. (2014) Dietary intervention modulates the gut microbiota and improves insulin resistance－a randomized controlled trial in obese postmenopausal women. Obesity review 15: 33-52.

10. Sonestedt E, Orho-Melander M (2010) High intake of fermented milk is associated with decreased risk of type 2 diabetes and better insulin sensitivity. Diabetologia 53: 358. **(Conference literature of EASD Annual Meeting)**

**Not RCT (9):**

1.Wong VWS, Wong GLH, Chim AML, Chu WCW, Yeung DKW, et al. (2013) Treatment of nonalcoholic steatohepatitis with probiotics. A proof-of-concept study. Annals of Hepatology 12: 256-262. **(This is a low quality RCT and is an open-label study)**

2. Abreu S, Moreira P, Moreira C, Mota J, Moreira-Silva I, et al. (2014) Intake of milk, but not total dairy, yogurt, or cheese, is negatively associated with the clustering of cardiometabolic risk factors in adolescents. Nutr Res 34: 48-57. **(Cross-sectional survey study)**

3. Schiffrin EJ, Parlesak A, Bode C, Bode JC, Van'T HMA, et al. (2009) Probiotic yogurt in the elderly with intestinal bacterial overgrowth: Endotoxaemia and innate immune functions. British Journal of Nutrition 101: 961-966. **(Cross-sectional survey study)**

4. Struijk EA, Heraclides A, Witte DR, Soedamah-Muthu SS, Geleijnse JM, et al. (2013) Dairy product intake in relation to glucose regulation indices and risk of type 2 diabetes. Nutr Metab Cardiovasc Dis 23: 822-828.**（This is a Danish population-based lifestyle intervention study）**

5.Larsen N, Vogensen FK, Van Den Berg FWJ, Nielsen DS, Andreasen AS, et al. (2010) Gut microbiota in human adults with type 2 diabetes differs from non-diabetic adults. PLoS ONE 5. **(Case-control study)**

6. Le K. A, Li Y, Xu X, Yang W, Liu T, et al. (2013) Alterations in fecal Lactobacillus and Bifidobacterium species in type 2 diabetic patients in Southern China population. Frontiers in Physiology 3 JAN. **(Case-control study)**

7. Gea IL, Gea AL, Ortuno MIQ, Siguero JPL, Urda A, et al. (2013) Gut microbiota in children with type 1 diabetes differs from healthy children: A case-control study. Hormone Research in Paediatrics 80: 131. **(Case-control study)**

8. Mikelsaar M, Stsepetova J, Hutt P, Kolk H, Sepp E, et al. (2010) Intestinal Lactobacillus sp. is associated with some cellular and metabolic characteristics of blood in elderly people. Anaerobe 16: 240-246. **(Cross-sectional survey study)**

9. Dougkas A, Minihane AM, Givens DI, Reynolds CK, Yaqoob P (2012) Differential effects of dairy snacks on appetite, but not overall energy intake. Br J Nutr 108: 2274-2285. **(This is a randomised within-subject experimental study and each subject was repeated test for 4 times)**

**Unsuitable intervention group (6):**

1. Chang BJ, Park SU, Jang YS, Ko SH, Joo NM, et al. (2011) Effect of functional yogurt NY-YP901 in improving the trait of metabolic syndrome. European Journal of Clinical Nutrition 65: 1250-1255. **（The comparison is between different functional ingredients of probiotics）**

2. Hendrixon V, Chomanskis Z, Balciunas L, Bagdonaite L, Kucinskiene Z (2008) Effect of sour milk enriched with conjugated linoleic acid and probiotics on the concentrations of oxidized low density lipoproteins and glucose in the blood plasma of young people. Pharmacologyonline 2: 839-844.

**(The comparison is between different ingredients of sour milk)**

3. Dugan CE, Barona J, Fernandez ML (2014) Increased dairy consumption differentially improves metabolic syndrome markers in male and female adults. Metabolic Syndrome and Related Disorders 12: 62-69 (**The comparison did not focus on probiotics)**

4. Bonet SB, Quintanar RA, Viana AM, Iglesias-Gutierrez E, Varela-Moreiras G (2008) The effects of yogurt with isomer enriched conjugated linoleic acid on insulin resistance in obese adolescents. Revista Espanola de Pediatria 64: 94-100. **（The comparison is between different functional ingredient (conjugated linoleic acid) in yogurt）**

5.Neyestani TR, Nikooyeh B, Alavi-Majd H, Gharavi A, Kalayi A, et al. (2011) Daily consumption of vitamin D-fortified yogurt drink both improved glycemic control and decreased serum cardiac myeoloperoxidase in type 2 diabetics. Atherosclerosis Supplements 12: 153. （**The comparison is between whether containing vitamin D-fortified supplement in yogurt drink**）

6. Rajala SA, Salminen SJ, Seppanen JH, Vapaatalo H (1988) Treatment of chronic constipation with lactitol sweetened yoghurt supplemented with guar gum and wheat bran in elderly hospital in-patients. Comprehensive gerontology. Section A, Clinical and laboratory sciences 2: 83-86. **（The comparison is between whether containing lactitol, guar gum and wheat bran in yoghurt）**

**Combined prebiotic (n=5)**

1. Asemi Z, Khorrami-Rad A, Alizadeh SA, Shakeri H, Esmaillzadeh A (2014) Effects of synbiotic food consumption on metabolic status of diabetic patients: a double-blind randomized cross-over controlled clinical trial. Clin Nutr 33: 198-203.

2. Moroti C, Magri LFS, Costa MDR, Cavallini DCU, Sivieri K (2012) Effect of the consumption of a new symbiotic shake on glycemia and cholesterol levels in elderly people with type 2 diabetes mellitus. Lipids in Health and Disease: 29.

3. Schaafsma G, Meuling WJA, Van Dokkum W, Bouley C (1998) Effects of a milk product, fermented by Lactobacillus acidophilus and with fructo-oligosaccharides added, on blood lipids in male volunteers. European Journal of Clinical Nutrition 52: 436-440.

4. Taghizadeh M, Asemi Z (2014) Effects of synbiotic food consumption on glycemic status and serum hs-CRP in pregnant women: A randomized controlled clinical trial. Hormones 13: 398-406.

5. Eslamparast T, Poustchi H, Zamani F, Sharafkhah M, Malekzadeh R, et al. (2014) Synbiotic supplementation in nonalcoholic fatty liver disease: A randomized, double-blind, placebo-controlled pilot study. American Journal of Clinical Nutrition 99: 535-542.

**Not continuous intervention (3):**

14. Mazloom Z, Yousefinejad A, Dabaghmanaesh MH (2013) Effect of probiotics on lipid profile, glycemic control, insulin action, oxidative stress, and inflammatory markers in patients with type 2 diabetes: A clinical trial. Iranian Journal of Medical Sciences 38: 38-43. **(Probiotics was one-time intervention)**

15. Sanggaard KM, Holst JJ, Rehfeld JF, Sandstrom B, Raben A, et al. (2004) Different effects of whole milk and a fermented milk with the same fat and lactose content on gastric emptying and postprandial lipaemia, but not on glycaemic response and appetite. British Journal of Nutrition 92: 447-459. **(Probiotics was one-time intervention)**

16. Maioli M, Pes GM, Sanna M, Cherchi S, Dettori M, et al. (2008) Sourdough-leavened bread improves postprandial glucose and insulin plasma levels in subjects with impaired glucose tolerance. Acta Diabetologica 45: 91-96. **(Probiotics was one-time intervention)**

**Not human (n=3)**

1. Amar J, Chabo C, Waget A, Klopp P, Vachoux C, et al. (2011) Intestinal mucosal adherence and translocation of commensal bacteria at the early onset of type 2 diabetes: Molecular mechanisms and probiotic treatment. EMBO Molecular Medicine 3: 559-572.

2. Murphy EF, Cotter PD, Hogan A, O'Sullivan O, Joyce A, et al. (2011) The gut microbiota - A realistic therapeutic target in obesity and metabolic dysregulation. Gastroenterology 140: S103-S104.

3. Wolf KJ, Lorenz RG (2010) Dietary alteration of intestinal microbiota alters incidence of type 1 diabetes. Diabetes.

**Not adult (n=2)**

1. Gobel RJ, Larsen N, Jakobsen M, Christian M, Michaelsen, KF (2012) Probiotics to adolescents with obesity: effects on inflammation and metabolic syndrome. Journal of pediatric gastroenterology and nutrition 55:673–678

2. Ljungberg M, Korpela R, Ilonen J, Ludvigsson J, Vaarala O (2006) Probiotics for the prevention of beta cell autoimmunity in children at genetic risk of type 1 diabetes--the PRODIA study. Ann N Y Acad Sci 1079: 360-364.

**Case-report (n=1):**

1. Rozanova GN, Voevodin DA (2008) [A case of an effective application of probiotics in the complex therapy of severe type 1 diabetes mellitus and intestinal disbacteriosis]. Klin Med (Mosk) 86: 67-68.

**No dose (n=2):**

1. Hove K, Brons C, Faerch K, Lund SS, Rossing P, et al. (2014) Effects of 12 weeks treatment with fermented milk on blood pressure, glucose metabolism and markers of cardiovascular risk in patients with type 2 diabetes: a randomised double-blind placebo-controlled study. Eur J Endocrinol.

2.Malaguarnera M, Vacante M, Antic T, Giordano M, Chisari G, et al. (2012) Bifidobacterium longum with fructo-oligosaccharides in patients with non alcoholic steatohepatitis. Digestive Diseases and Sciences 57: 545-553.

**Unfinish (n=1):**

1. Nitert MD, Barrett HL, Foxcroft K, Tremellen A, Wilkinson S, et al. (2013) SPRING: an RCT study of probiotics in the prevention of gestational diabetes mellitus in overweight and obese women. BMC Pregnancy Childbirth 13: 50. **(This is a protocol and the study has not been finished before we searched)**

**Not English (n=2):**

1. F S, CG Y, X L, DG Y (2012) Application of microbiological and immunological enteral nutrition in patients with gastrointestinal cancer complicated with diabetes mellitus. Chinese Journal of Gastrointestinal Surgery.

2. Bayat A, Heydaribeni M, Feizi A, Iraj B, Ghiasvand R, et al. (2014) The effect of pumpkin and probiotic yogurt consumption separately or/and simultaneously on type II diabetes. Journal of Isfahan Medical School 32: 580-589.
