# Supplementary material for: Effect of Probiotics on Glycemic Control: A Systematic Review and Meta-Analysis of Randomized, Controlled Trials
Source: PLoS One. 2015 Jul 10;10(7):e0132121. doi: 10.1371/journal.pone.0132121 (PMC4498615; doi:10.1371/journal.pone.0132121)
Supplement: S1 Table — (DOCX) [file pone.0132121.s007.docx]

**Table 3. Sensitivity analysis of RCTs**

| ***Groups*** | ***Trials*** | | ***WMD*** | ***95% CI*** | ***P*** | | | ***I^2^ (%)*** | | ***P _heterogeneity_*** |
| --- | --- | --- | --- | --- | --- | --- | --- | --- | --- | --- |
| Blood Glucose (mmol/L) | | | | | | | | | | |
| Excluded heterogeneous studies | 13 | | -0.16 | -0.23, -0.09 | | <0.01 | | 31 | | 0.13 |
| Studies with sample size ≥ 20 | 9 | | -0.18 | -0.35, -0.01 | | 0.04 | | 53 | | 0.03 |
| Insulin (μU/ml) | | | | | | | | | | |
| Excluded heterogeneous studies | 7 | -1.17 | | -1.48, -0.87 | | | <0.01 | | 0 | 0.66 |
| Studies with sample size ≥ 20 | 8 | -1.09 | | -2.37, 0.19 | | | 0.09 | | 90 | <0.01 |
| HOMA-IR | | | | | | | | | | |
| Excluded heterogeneous studies | 3 | -0.31 | | -0.57, -0.05 | | | 0.02 | 32 | | 0.22 |
| Studies with sample size ≥ 20 | 6 | -0.42 | | -0.94， 0.01 | | | 0.12 | 95 | | <0.01 |

WMD, weight mean difference.
